# Rutin is a potent senomorphic agent to target senescent cells and can improve chemotherapeutic efficacy

**DOI:** 10.1111/acel.13921

**Published:** 2023-07-20

**Authors:** Hanxin Liu, Qixia Xu, Halidan Wufuer, Zi Li, Rong Sun, Zhirui Jiang, Xuefeng Dou, Qiang Fu, Judith Campisi, Yu Sun

**Affiliations:** ^1^ Department of Pharmacology Institute of Aging Medicine, Binzhou Medical University Yantai China; ^2^ CAS Key Laboratory of Tissue Microenvironment and Tumor Shanghai Institute of Nutrition and Health, Chinese Academy of Sciences Shanghai China; ^3^ Shanghai Institute of Nutrition and Health, Chinese Academy of Sciences Shanghai China; ^4^ Department of Discovery Biology Bioduro‐Sundia, Zhangjiang Hi‐Tech Park Shanghai China; ^5^ Buck Institute for Research on Aging Novato California USA; ^6^ Lawrence Berkeley National Laboratory University of California Berkeley California USA; ^7^ Department of Medicine and VAPSHCS University of Washington Seattle Washington USA

**Keywords:** age‐related pathology, aging, ASAP, cellular senescence, chemotherapy, rutin, SASP, senomorphics

## Abstract

Aging is a major risk factor for most chronic disorders, for which cellular senescence is one of the central hallmarks. Senescent cells develop the pro‐inflammatory senescence‐associated secretory phenotype (SASP), which significantly contributes to organismal aging and age‐related disorders. Development of senotherapeutics, an emerging class of therapeutic agents to target senescent cells, allows to effectively delay aging and alleviate chronic pathologies. Here we report preliminary outputs from screening of a natural medicinal agent (NMA) library for senotherapeutic candidates and validated several agents with prominent potential as senomorphics. Rutin, a phytochemical constituent found in a number of plants, showed remarkable capacity in targeting senescent cells by dampening expression of the full spectrum SASP. Further analysis indicated that rutin restrains the acute stress‐associated phenotype (ASAP) by specifically interfering with the interactions of ATM with HIF1α, a master regulator of cellular and systemic homeostasis activated during senescence, and of ATM with TRAF6, part of a key signaling axis supporting the ASAP development toward the SASP. Conditioned media produced by senescent stromal cells enhanced the malignant phenotypes of prostate cancer cells, including in vitro proliferation, migration, invasion, and more importantly, chemoresistance, while rutin remarkably downregulated these gain‐of‐functions. Although classic chemotherapy reduced tumor progression, the treatment outcome was substantially improved upon combination of a chemotherapeutic agent with rutin. Our study provides a proof of concept for rutin as an emerging natural senomorphic agent, and presents an effective therapeutic avenue for alleviating age‐related pathologies including cancer.

## Graphical abstract

Senescent cells in solid organs are a major driving force of chronological aging and various age‐related pathologies. In the early stage of the SASP development (ASAP), ATM physically interacts with HIF1a and TRAF6. Rutin, a natural phytochemical agent, can impede the interaction between ATM and HIF1a or TRAF6. Through effectively suppressing the SASP development, rutin holds a substantial value to serve as a novel senomorphic agent in future clinics.

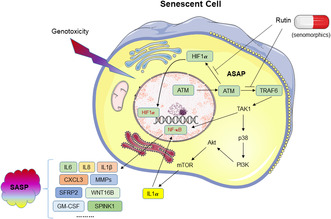

## 1 INTRODUCTION

Aging is accompanied by a progressive decline of organ functions and the inability to respond to environmental stresses. The hallmarks of aging include genomic instability, epigenetic changes, mitochondrial dysfunction, stem cell exhaustion, and other abnormalities that together contribute to the loss of tissue homeostasis, cause age‐related disorders and increase overall morbidity (Lopez‐Otin et al., 2013; Partridge et al., 2020). Cellular senescence is a typical hallmark of organismal aging and promotes multiple chronic pathologies, including but not limited to neurodegenerative diseases, cardiovascular disorders, and various types of malignancies (Kirkland & Tchkonia, 2020; Song, Lam, et al., 2020; Song, Tchkonia, et al., 2020; Sun et al., 2022). Recent advances supporting the causal role of cellular senescence in diverse age‐associated conditions highlight the potential of targeting senescent cells as an efficient therapeutic strategy to prevent multiple age‐associated symptoms, namely comorbidity, and to extend human health span (Xu et al., 2018; Xu, Fu, et al., 2021).

The therapeutic benefits of selectively targeting senescent cells with pharmacological agents, now generally termed senotherapeutics, have been demonstrated by an increasing number of studies. There are normally two categories of senotherapeutics: senolytics, which induce programed death of senescent cells, and senomorphics, which curtail the pro‐inflammatory senescence‐associated secretory phenotype (SASP) to cause senostasis (Song, Lam, et al., 2020). The majority of the senolytics identified to date induce apoptosis of senescent cells by targeting key enzymes involved in prosurvival and antiapoptotic mechanisms, such as BCL‐2 family members (Yosef et al., 2016; Zhu et al., 2015, 2016, 2017). By contrast, senomorphics provides an alternative approach to intervene senescence by suppressing detrimental effects of the SASP without eliminating these cells. Of note, the early senomorphics were largely discovered by serendipity, including aspirin, metformin, rapamycin, and resveratrol (Zhang et al., 2022).

Some natural products, especially polyphenols and flavonoids, exhibit remarkable antioxidant and anti‐inflammatory activities, holding the potential to target cellular senescence and/or suppress the inflammatory SASP. Flavonoids such as apigenin and kaempferol can effectively inhibit the SASP in senescent human fibroblasts generated by chemotherapeutic or hydrogen peroxide (H_2_O_2_) treatment, thus considered as promising phytochemicals to minimize the impact of senescent cells in age‐related conditions (Li et al., 2021; Lim et al., 2015). In the case of idiopathic pulmonary fibrosis (IPF), quercetin restores the susceptibility of senescent fibroblasts to proapoptotic stimuli, reverses pulmonary fibrosis, and prevents weight loss by increasing the expression of FasL receptor and caveolin‐1 (Hohmann et al., 2019). However, many natural products are notoriously known for their complex modes of action with multiple targets, while poor bioavailability remains as a common concern for many polyphenol natural products.

As a natural flavonoid, rutin is known as quercetin‐3‐O‐rutinoside and vitamin P. Specifically, rutin is a lipophilic ingredient, making it soluble in organic solvents such as pyridine, methanol, and ethanol (Negahdari et al., 2021). However, it has relatively limited natural stability and bioavailability, physicochemical properties mainly due to its low solubility in water (Gullon et al., 2017). Rutin can be readily found in vegetables, citrus fruits, and plant‐derived beverages. Effects generated by the reducing properties of rutin on different oxidizing species such as superoxide, peroxyl, and hydroxyl radicals provide substantial antioxidant benefits (Mauludin et al., 2009). It also displays pharmacological activities such as antimicrobial and anti‐inflammatory capacities (Panasiak et al., 1989), although sometimes showing counteractive activity against certain other flavonoids which exert pro‐oxidant activities by catalyzing oxygen radical generation (Hodnick et al., 1986). To date, rutin has been considered as a nontoxic chemical, which may be useful in numerous biomedical applications, while some rutin‐containing natural plant extracts such as Ginkgo biloba extract (GBE) demonstrated vital health benefits such as those observed in alleviation of chronic neurodegenerative diseases and reduction in extracellular amyloid beta (Aβ) plaques, typical symptoms of Alzheimer's disease (AD) (Morato et al., 2023). By contrast, the potential of rutin in other situation, particularly those involving antiaging properties in advanced stage and the potential of using its bioactivity in development of senotherapeutics, more specifically senomorphics, remains largely underexplored. In this work, we screened a library of natural medicinal agents, and noticed the emerging value of rutin in targeting human senescent cells. Further assessments disclosed its functional mechanism and pharmacological value in modulation of senescence‐associated phenotypes, suggesting its potential benefits in governing the activities of senescent cells and possible contributions in future geriatric medicine.

## 2 RESULTS

### 2.1 Drug screening reveals rutin as a potential senomorphic agent

**FIGURE 1:**
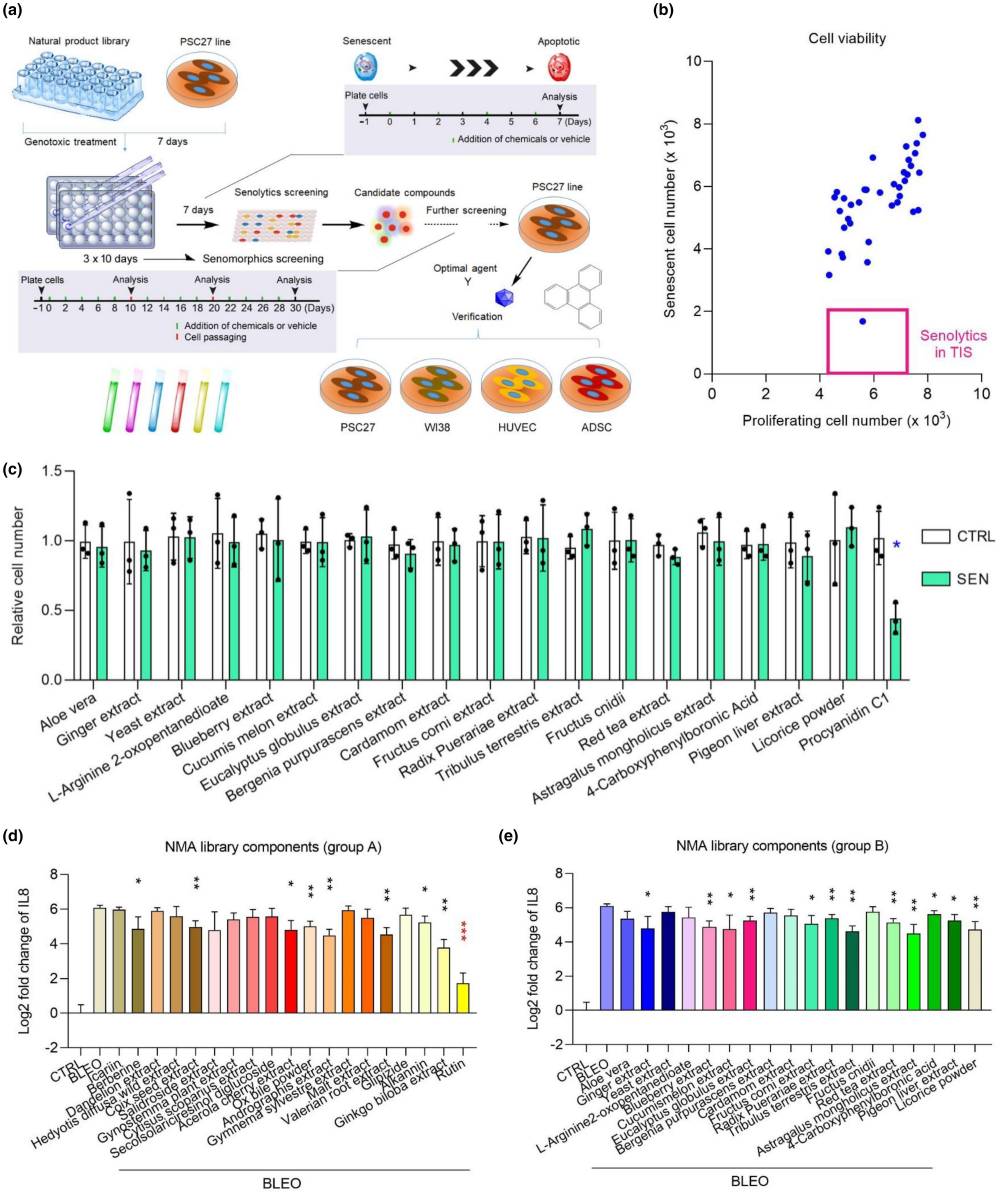
In vitro screening of a natural medicinal agent (NMA) library for senotherapeutic candidates. (a) A schematic workflow of cell‐based screening strategy for the NMA library, which comprises 37 natural medicinal agents. After the 1st round of senolytics screening was complete, all candidate agents were subject to the 2nd round of senomorphics screening. The efficacy of potent candidates was subsequently validated in multiple stromal cell lines of human origin. (b) Preliminary screening outputs after in vitro pharmacological assays. Library agents were each assessed at a concentration of 3 μg/mL with 5.0 × 10^3^ cells for 3 d. Hits were selected based on their ability to specifically kill senescent rather than control cells. Each blue dot represents a single agent as the mean of three replicates. Red rectangular region denotes potential candidates of senolytics in the case of TIS. Note, only the positive control, procyanidin C1 (PCC1), fell within this region. (c) Assessment of the effects of individual agents (herein group B) on the survival of CTRL and SEN cells in culture. (d) Appraisal of the effects of natural agents (19 candidates in the library, as group A, each applied at 1 μg/mL) on the expression profile of human genes, and more specifically, of the typical SASP factor IL8, in CTRL and SEN cells. (e) Appraisal of the effects of natural agents (the other 18 candidates in the library, together as group B, each applied at 1 μg/mL) on the expression profile of typical SASP factor IL8. The graphic design of (a) referred to a former literature reporting the screening results of geroprotective compounds (Geng et al., 2019), and specifically managed to highlight the difference between senescent and apoptotic cells. For all datasets, samples were collected for analyses 3 days after treatment with individual agents in culture condition. CTRL, control; SEN, senescent; TIS, therapy‐induced senescence. Data in b, c, d and e are shown as mean ± SD and representative of three independent biological replicates, with *p* values calculated by a two‐sided *t* test. *p < 0.05; **p < 0.01. ***p < 0.001. [Correction added on 17 Aug 2023, after first online publication: New reference (Geng et al, 2019) has been added in the reference list and the legend of figure 1 has been revised.]

To identify new compounds that can effectively target senescent cells, we performed an unbiased agent screening with a library composed of 37 natural medicinal agents (NMAs), most of which are phytochemical products (Table S1). To this end, a primary normal human prostate stromal cell line, PSC27, was selected as a cell‐based model. Comprising mainly of fibroblasts but with a minor percentage of nonfibroblast cell lineages such as smooth muscle cells and endothelial cells, PSC27 is primary per se and develops a typical SASP upon exposure to stressors such as genotoxic chemotherapy and ionizing radiation (Han et al., 2020; Sun et al., 2012; Xu et al., 2019; Zhang et al., 2018). To induce senescence, we treated cells with a preoptimized sublethal dose of bleomycin (BLEO) (50 μg/mL) and observed elevated positivity of senescence‐associated β‐galactosidase (SA‐β‐Gal) staining, reduced BrdU incorporation and augmented DNA damage response (DDR) foci 7–10 days afterwards (Figure S1a–c). We set up a screening strategy to compare the effect of individual medicinal products generated on the survival and expression profile of senescent cells (Figure 1a).

We first determined the efficacy of these NMA components against senescent PSC27, and explored its potential as an experimental cell model for overall drug screening. Our preliminary data suggested that a number of these compounds were able to alter the bioactivity of senescent, but not proliferating cells (Figure 1b–e) and (Figure S1d). Such a property implied that PSC27 represents an ideal and qualified model to initiate subsequent procedures, as it allows to selectively target senescent cell populations rather than their growing counterparts, largely securing the feasibility of using this primary stromal line for further investigations. Upon large scale screening of the NMA library, we failed to identify even a single senolytic agent, which was supposed to hold the potential to selectively kill senescent cells in culture like the positive control procyanidin C1 (Figure 1b,c and Figure S1d), suggesting the scarcity of such natural senolytics and difficulty in expanding the arsenal of this specific subclass of senotherapeutics. However, we observed that a handful of NMA components exhibited a remarkable senomorphic potential, which did deserve continued investigations (Figure 1d,e).

Among the senomorphic agents that showed prominent efficacy in downregulating the expression of interleukin 8 (IL8), a hallmark SASP factor, we selected rutin for analysis in depth (Figure S1e,f). As a flavonoid derived from wildlife plants, rutin has been reported with a wide range of biological activities including anti‐inflammatory, antioxidant, neuroprotective, and hepatoprotective effects (Ghorbani, 2017). Rutin restrains the production of reactive oxygen species (ROS), advanced glycation end product precursors, and inflammatory cytokines, effects considered responsible for the protective effect of rutin against several pathological conditions such as hyperglycemia, nephropathy, neuropathy, and cardiovascular disorders. However, its medical implications including therapeutic capacity and functional mechanisms, in modulating the activities of senescent cells, particularly the SASP, remain generally unknown.

### 2.2 Rutin dampens expression of the full spectrum SASP without affecting cellular senescence

**FIGURE 2:**
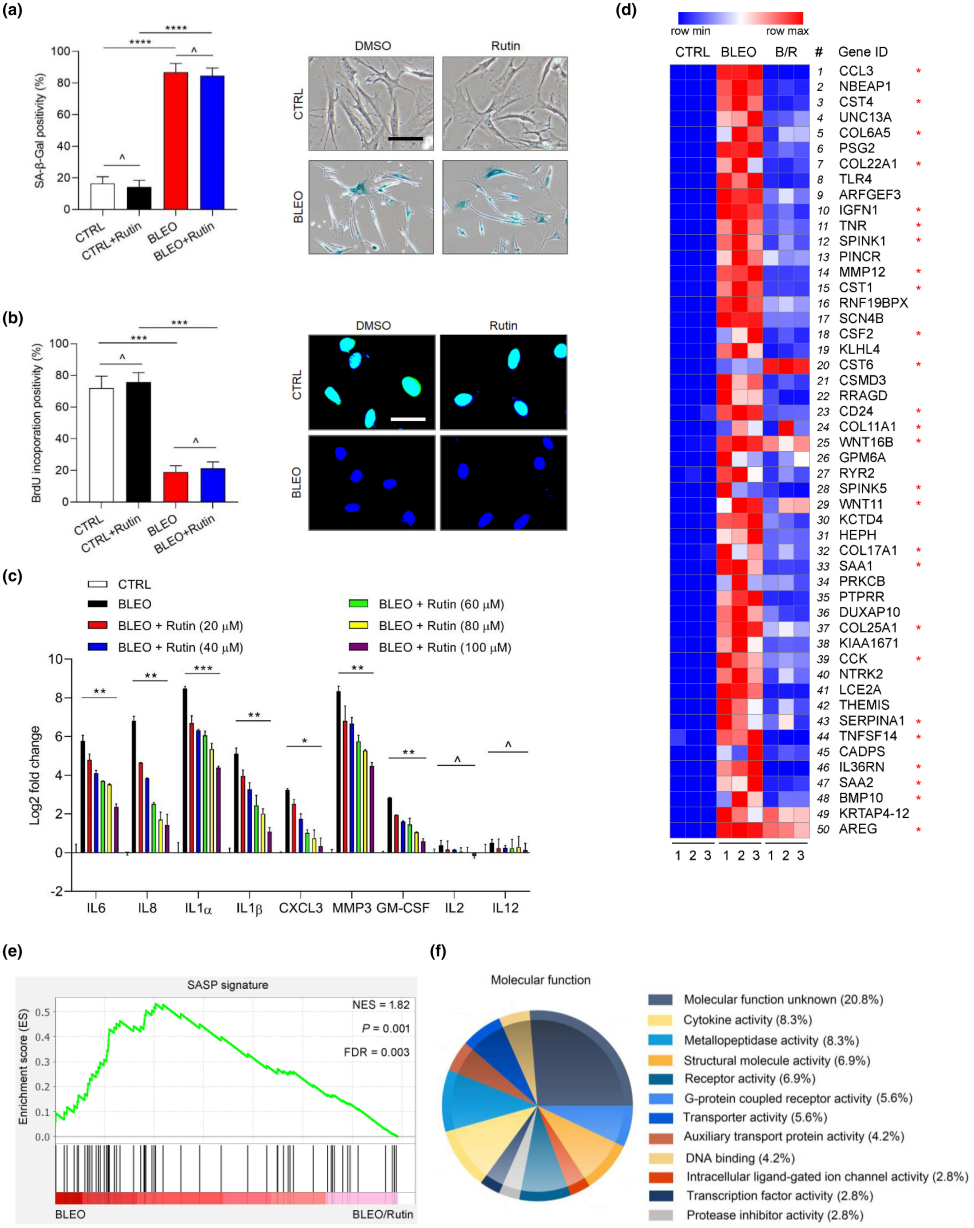
Analysis of the effects of rutin on cellular senescence and expression profile of senescent cells. (a) Evaluation of cellular senescence by SA‐β‐Gal staining. Left, comparative statistics. Right, representative images of SA‐β‐Gal staining. Scale bar, 20 μm. (b) Evaluation of cell cycle arrest by BrdU staining. Left, comparative statistics. Right, representative images of BrdU staining. Scale bar, 15 μm. (c) Quantitative measurement of typical SASP factor expression at transcription level upon treatment of BLEO‐induced senescence with or without rutin in culture. (d) Heatmap depicting the expression landscape of PSC27 cells upon senescence induction and/or rutin intervention. Human genes were ordered according to their upregulation fold change in control versus senescent cells, with corresponding changes in the presence of rutin displayed in parallel. Rutin was applied at a concentration of 100 μM. (e) GSEA plot presentation of significant gene set indicative of the development of a typical SASP. (f) A pie chart displaying the molecular function (MF) of top 100 genes that were most downregulated upon treatment by rutin in senescent PSC27 cells. For all datasets, cells were collected for analyses 7 days after BLEO treatment or 3 days after rutin treatment of senescent cells in culture condition. Data in a–c are representative of three independent biological replicates. *p* values calculated by a two‐sided *t* test. ^*p* > 0.05; *p < 0.05; **p < 0.01; ***p < 0.001; ****p < 0.0001.

Although rutin inhibits expression of the SASP hallmark factor IL8, whether it affects cellular senescence and a wide spectrum of the SASP, or alternatively, the vast majority of other SASP factors, remains yet unclear. To address these questions, we performed in vitro assays and found that SA‐β‐Gal staining and BrdU incorporation remained largely unchanged, regardless of proliferating or senescent cells, the latter induced by bleomycin (BLEO), a genotoxic agent frequently administered to cancer patients in clinical oncology (Figure 2a,b).

Among the different concentrations we used in culture, 100 μM of rutin seemed to have generated the most optimal effects in restraining the expression of a subset of typical SASP components, including IL6, IL8, IL1α, IL1β, CXCL3, MMP3, and GM‐CSF (Figure 2c). We also examined the expression of IL2 and IL12, two pro‐inflammatory cytokines that are typically not associated with the SASP. The results indicated that expression of neither of these factors was altered in response to treatment by BLEO, rutin or cotreatment by both agents, substantiating their non‐SASP nature (Figure 2c). Moreover, analysis of RNA‐seq datasets to profile the transcriptome‐wide expression pattern of PSC27 indicated that most of the SASP factors were indeed downregulated by rutin, although the expression status of some genes not directly correlated with cellular senescence or the SASP was also altered (Figure 2d). As supporting evidence, GSEA mapping outputs largely confirmed the influence of rutin on senescent cell expression, suggesting a specifically and significantly inhibited SASP (Figure 2e).

Further analysis by bioinformatics revealed that 4619 transcripts were significantly modified (2‐fold change of log2‐based, *p* < 0.01) in senescent PSC27 cells upon exposure to rutin in the culture condition (3733 downregulated and 886 upregulated) (Figure S2a). After mapping the transcripts to a gene ontology (GO) database comprising HPRD, Entrez Gene, and UniProt accession identifiers (Keshava Prasad et al., 2009; Maglott et al., 2011; UniProt, 2010), we noticed that the most prevalent molecular functions of upregulated genes (top 100 selected as representatives) were cytokine activity, metallopeptidase activity, structural molecule activity, and receptor activity (Figure 2f). The most typical cellular components of bioactive proteins encoded by these genes were those transported to extracellular niche, followed by those residing in the cytoplasm and nucleus, although many products indeed belong to the subcategories of soluble fraction, extracellular space, extracellular region, and exosomes (Figure S2b). In addition, the most predominant biological processes correlated with the upregulated genes were intercellular communication, inflammatory response, cell growth and/or maintenance, regulation of nucleobase, nucleoside, nucleotide, and nucleic acid metabolism (Figure S2c). Together, our data suggest a salient capacity of rutin in restraining the expression of genes closely correlated with pro‐inflammatory response and secretory activity of senescent cells, or alternatively, falling in the range of the SASP spectrum.

To expand and confirm the reproducibility of experimental data, we employed alternative approaches to induce senescence, including replicative senescence (RS) and oncogene‐induced senescence (OIS). The data suggest that rutin can consistently downregulate the expression of most of the representative SASP factors in senescent cells, regardless of senescence induction modality (Figure S2d,e). In addition, we repeated the assays with WI38 and IMR90, two typical human stromal cell lines frequently used in senescence and aging studies, resulting in the acquisition of datasets largely resembling those of PSC27 (Figure S2f,g). Thus, rutin widely suppresses the SASP expression independent of senescence induction method and target cell line.

### 2.3 Rutin inhibits the ASAP by interfering with the interactions of ATM with HIF1α and TRAF6

**FIGURE 3:**
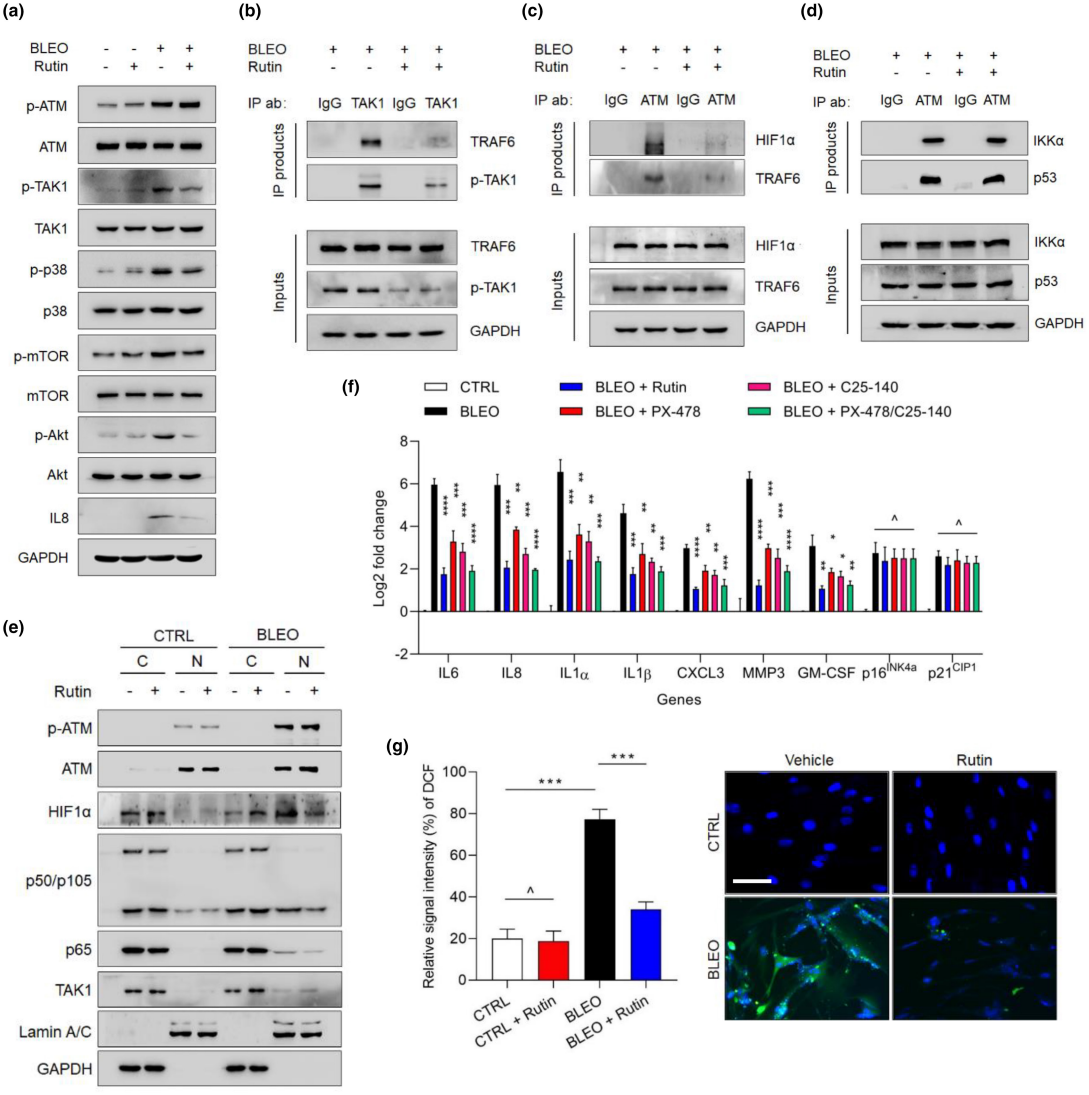
Determination of the molecular and cellular mechanisms allowing rutin to restrain the SASP in a senescence setting. (a) Immunoblot analysis of key factors involved in activation of pathways that are essential for the SASP development. Cellular senescence was induced by treatment with BLEO. GAPDH, loading control. (b) Immunoprecipitation (IP) followed by immunoblot assay with the whole lysates of stromal cells. PSC27 was treated by BLEO for 12 h before change of media, and cells were allowed to senesce in culture for 7 d prior to cell lysis. Alternatively, rutin was added to the media and cells were lysed 3 d afterwards. Antibodies including IgG and anti‐TAK1 were used for IP, with TRAF6 and p‐TAK1 in both IP products and inputs analyzed. (c) IP followed by immunoblot assessment with the whole lysates of stromal cells. Cells were treated as described in (b). Antibodies including IgG and anti‐ATM were used for IP, with HIF1α and TRAF6 in both IP products and inputs analyzed. (d) An IP/immunoblot assay similar with that described in (c). Antibodies including IgG and anti‐ATM were used for IP, with IKKα and p53 in both IP products and inputs analyzed. (e) Immunoblot analysis of ATM, HIF1α, p50, and p65 nuclear translocation in CTRL and BLEO‐treated PSC27 cells. C, cytoplasmic; N; Nuclear. Lamin A/C and GAPDH, loading control for nuclear and cytoplasmic proteins, respectively. (f) Quantitative assessment of the expression of a subset of typical SASP factors and senescence biomarkers at transcription level upon treatment of BLEO‐induced senescence. Rutin, PX‐478 and C25‐140 were applied to treat cells in culture. (g) Measurement of ROS with 2′‐7′‐dichlorodihydrofluorescein diacetate (DCFH‐DA), a cell‐permeable fluorescent probe sensitive to changes in cellular redox state. Experiments were performed 1 d after rutin treatment. Left, comparative statistics. Scale bar, 20 μm. Right, representative images. Data in a–g are representative of three independent biological replicates. *p* values calculated by a two‐sided *t* test. ^*p* > 0.05; *p < 0.05; **p < 0.01; ***p < 0.001; ****p < 0.0001.

We next questioned the molecular mechanism supporting rutin to generate influence on the development of cellular senescence‐associated phenotypes, particularly the SASP. To assess the potential actions of rutin on intracellular DDR signaling, an event that allows genomic DNA damage to activate downstream inflammatory responses, we first collected total lysates of PSC27 cells before and after rutin treatment of proliferating or senescent cells. Immunoblot indicated that ATM, one of the central regulators of DDR signaling, was markedly activated upon induction of senescence by BLEO (Figure 3a). In response to inherent or environmental stimuli, proliferating cells tend to firstly exhibit an acute stress‐associated phenotype (ASAP), which involves prompt ATM activation and nucleus‐to‐cytoplasm translocation, TRAF6‐mediated mono‐ubiquitylation and TAK1 phosphorylation, a process that can be observed within 2–3 days after exposure of cells to insulting damage (Zhang et al., 2018). As an essential component of genotoxicity stress‐induced cellular responses and a key modulator of the ASAP, the cytoplasmic kinase TAK1 was clearly phosphorylated in our assays, a change functionally priming it for subsequent engagement in dual feedforward mechanisms to orchestrate the SASP development (Zhang et al., 2018). We further noticed that activation of p38MAPK, a downstream target of TAK1, activation of the PI3K/Akt/mTOR axis (mediator of the persistent SASP signaling, indicated by mTOR and Akt phosphorylation), as well as upregulation of IL8, the hallmark factor for ASAP (also for SASP, in most cell lines), took place in an acute manner after BLEO treatment (Figure 3a). However, in the presence of rutin, these remarkable changes generally diminished, except that ATM phosphorylation remained largely unaffected, suggesting that the potential target of rutin is likely downstream of ATM, but upstream of TAK1, p38MAPK, and other regulators, and such a target is functionally implicated in the acute response upon induction of cellular senescence (Figure 3a).

In the course of ASAP, there is a physical association between activated TRAF6 and TAK1, a phenomenon that shows up shortly after DNA damage but subject to suppression by the TAK1 inhibitor, 5*Z*‐7‐oxozeaenol (Zhang et al., 2018). However, whether rutin disrupts this process remains unknown. We thus chose to perform phosphorylated TAK1 (p‐TAK1)‐mediated IP and subsequent immunoblot analysis, and found that both TAK1 activation and TRAF6‐TAK1 interaction were considerably restrained by rutin (Figure 3b). The data confirmed that the direct target(s) rutin should be beyond the physical association of TRAF6 and TAK1, although further validation is needed to support this hypothesis.

To establish the mechanism responsible for implications of rutin in the ASAP process, the early response that eventually culminates in the SASP formation as a chronic phenomenon, we performed genome‐wide mapping of molecules that hold the potential to interact with ATM. Bioinformatics mining revealed 279 unique ATM interactors and 379 unique TRAF6 interactors in human cells, with 22 interacting molecules shared by both ATM and TRAF6 in the final outputs (Table S2 and Figure S3a,b). Further analysis excluded the necessity of exploring the vast majority of molecules that can interact with both ATM and TRAF6, with hypoxia‐inducible factor 1α (HIF1α) standing out as a candidate that deserves continued investigation.

Data from p‐ATM‐mediated IP and corresponding immunoblot analysis consolidated that ATM and TRAF6 can interact with each other, but rutin significantly weakens such an interaction. More importantly, there is a mutual interaction between ATM and HIF1α, which was also subject to interruption by rutin (Figure 3c). By contrast, rutin did not seem to hinder the association of ATM with IKKα (CHUK) or p53 (TP53), two randomly selected ATM interactor molecules as indicated by the resulting output of bioinformatics (totally 22), suggesting that rutin does not prevent the association of ATM with all interacting factors (Figure 3d). As further evidence, we observed remarkable cytoplasm‐to‐nucleus translocation of p65 and p50, two master subunits of NF‐κB transcriptional complex upon cellular senescence, although this tendency was largely abolished in the presence of rutin (Figure 3e). Of note, HIF1α exhibited nuclear translocation in a manner generally resembling that of p65 and p50 (Figure 3e), suggesting a potential engagement of this factor in modulating the genome‐wide expression of genes correlated with senescence and/or senescence‐associated phenotypes, specifically the SASP. Like the transcriptional complex NF‐κB, HIF1α is a key transcription factor for adaptive responses, orchestrating the transcription of numerous genes involved in angiogenesis, erythropoiesis, glycolytic metabolism, and inflammation (Yu et al., 2022), while its implication in senescence remains basically underexplored. HIF1α acts as a central factor that transmits ATM‐relayed damage signals to nucleus (Rezaeian et al., 2017; Tocci et al., 2023), and may be responsible for upregulation of a series of factors essential for senescence maintenance and the SASP development. To substantiate this speculation, we assessed the expression of a subset of genes encoding the SASP factors or senescence‐specific markers. Our data suggested that treatment with either PX‐478, a selective HIF‐1α inhibitor, or C25‐140, a small molecule compound that reduces TRAF6‐mediated ubiquitin chain formation, was able to significantly decrease the expression of typical SASP factors examined in the assay (Figure 3f). When both inhibitors were used, we observed further reduced expression of the SASP, with the fold change even approaching that caused by rutin alone. However, expression of p16^INK4a^ and p21^CIP1^ seemed to remain unaffected, suggesting the presence of differential regulatory pathways involved in upregulation of these factors upon cellular senescence.

To further consolidate the data, we functionally restored the pathway of HIF1α and/or TRAF6 to determine the functional implications of these molecules in SASP development. We found that restoration of either HIF1α or TRAF6 was not sufficient to resume the full SASP expression in rutin‐treated cells, but restoration of both pathways was able to largely rescue the SASP, overriding the effect of rutin (Figure S3c). Together, modulating the interactions between ATM and its key targets specifically HIF1α and TRAF6, as exemplified by the natural agent rutin, holds the potential to suppress the SASP expression, while sustaining cellular senescence, a distinct feature that is largely consistent with the criteria of senomorphics.

Since natural flavonoid polyphenols like rutin have antioxidant properties, decrease ROS production, and reduce oxidative stress, a phytochemical activity that can quickly arise under adverse conditions, particularly upon inherent and/or environmental insults, we asked whether similar effects could be observed in senescent cells exposed to rutin. In response to genotoxicity delivered by BLEO, PSC27 cells exhibited remarkably elevated ROS levels, in contrast to their normal counterparts (Figure 3g). Although rutin did not change the production of ROS in proliferating cells, it significantly suppressed the capacity of ROS generation by senescent cells, thus typically in line with its free radical scavenging ability.

### 2.4 Rutin deprives cancer cells of malignancy conferred by senescent stromal cells in a paracrine manner

**FIGURE 4:**
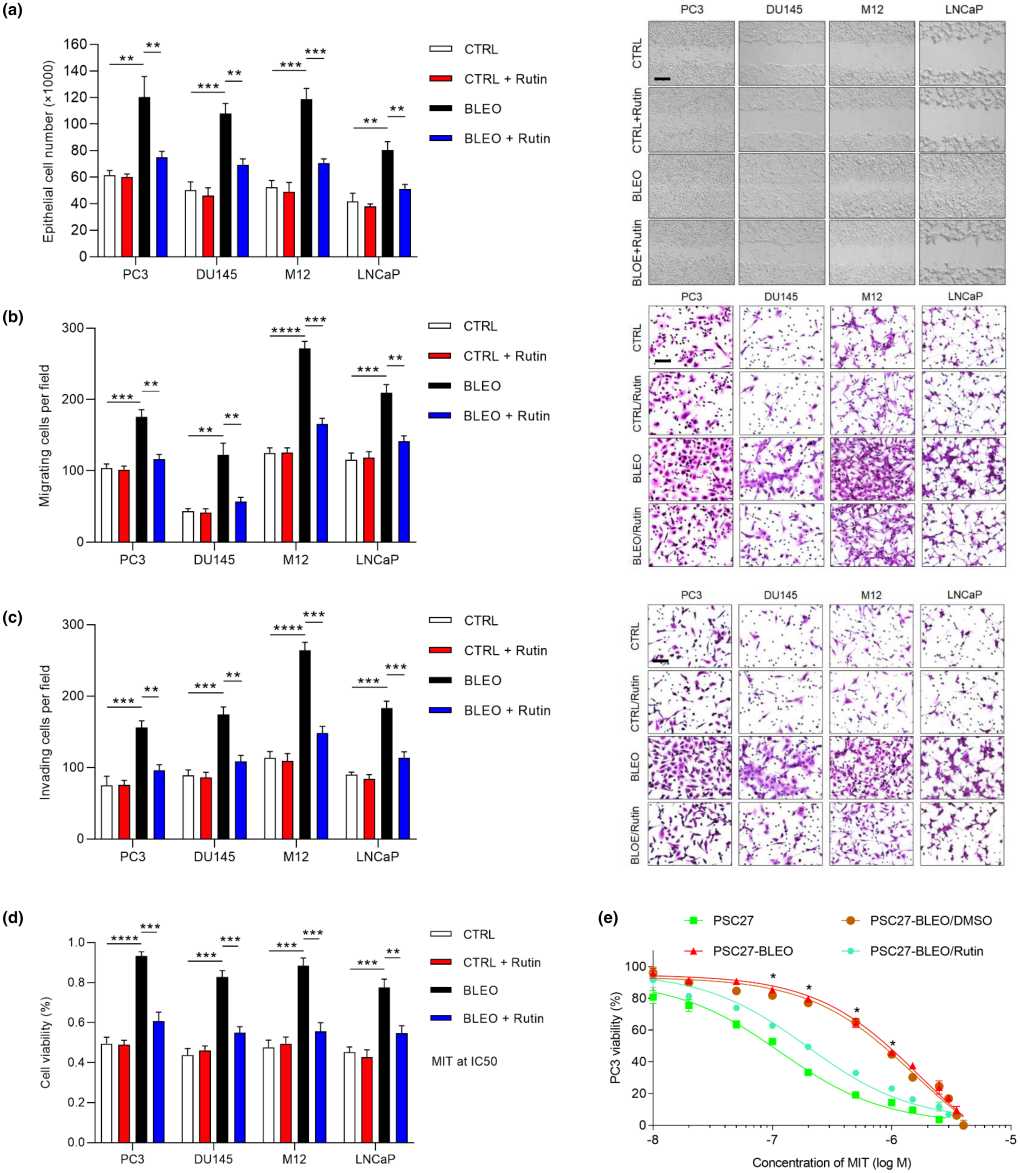
Rutin deprives prostate cancer cells of enhanced malignancies conferred by the conditioned media of senescent stromal cells. (a) PCa cells were treated with the conditioned media (CM) from PSC27 sublines as indicated for 3 d, and subject to cell proliferation assay. Native and senescent stromal cells generated by BLEO treatment were employed, with the CM collected 7 d after drug treatment and used for PCa cell culture (PC3, DU145, M12, and LNCaP). The CM were collected from equal number of cells *per* condition, with a starting DMEM that contains 0.5% FBS to make the CM. Left, comparative statistics. Right, representative images. (b) Migration assay of PCa cells described in (a) and seeded within trans‐wells in 6‐well plates, with cells cultured for 3 d in the CM from PSC27 sublines depicted in (a). (c) Invasiveness measurement of PCa cells described in (a) across the trans‐well membrane upon culture with the CM from PSC27 sublines described in (a). (d) Chemoresistance assay of PCa cells described in (a) cultured with the CM from PSC27 sublines described in (a). MIT (mitoxantrone) was applied at the concentration of IC50 value predetermined *per* cell line. (e) Dose–response curves (nonlinear regression/curve fit) plotted from drug‐based survival assays of PC3 cells cultured with the CM of PSC27 native or induced senescent by BLEO (PSC27‐BLEO), and concurrently treated by a wide range of concentrations rutin. In a–c, scale bars, 200 μm. Data are representative of three independent experiments. All *p* values were calculated by Student's *t* tests. *p < 0.05. **p < 0.01. ***p < 0.001. ****p < 0.0001.

Our previous studies reported the capacity of a full spectrum SASP in promoting the malignant phenotypes of cancer cells, and is one of major driving forces in tumor progression (Chen et al., 2018; Xu et al., 2019; Zhang et al., 2018). Here, we investigated the effect of rutin on human prostate cancer (PCa) cell behaviors by culturing with stroma cell‐derived conditioned media (CM) with and without rutin. Upon treatment with the CM from senescent PSC27 cells, not surprisingly, we observed substantially elevated proliferation of several PCa cell lines PC3, DU145, M12, and LNCaP (*p* < 0.01) (Figure 4a), accompanied by enhanced activities of migration and invasion (Figure 4b,c). However, these gain‐of‐functions almost completely disappeared upon rutin treatment of stromal cells (Figure 4a–c).

A number of SASP components including WNT16B, SFRP2, SPINK1, and AREG display strong capacity in conferring resistance to cancer cells (Chen et al., 2018; Sun et al., 2012, 2016; Xu et al., 2019). However, whether such a resistance‐promoting capacity of the SASP can be prevented by rutin, remains undetermined yet. We found the viability of cancer cells substantially increased upon exposure to senescent stromal cell‐derived CM, but counteracted by ~80% upon rutin application (Figure 4d). Although PSC27‐BLEO CM increased the viability of PC3 exposed to MIT at 0.1–1.0 μM, a range of doses approaching its serum concentrations in patients under clinical conditions (Costa et al., 2020; Yang et al., 2014), rutin was able to remarkably downregulate cancer resistance conferred by the CM of senescent stromal cells (Figure 4e). To exclude the potential contribution of enhanced cell proliferation on the observed cancer resistance, we chose to measure both activities in markedly shortened periods, such as 24 h, after experimental startup. Of note, PCa cells exhibited significantly increased resistance to the chemotherapeutic agent MIT, even when they did not gain elevated proliferative capacity yet at 24th h (Figure S4a,b). However, drug resistance of these cells was still markedly weakened in the presence of rutin. Therefore, our data suggest that cancer cells hold the potential to develop chemotherapeutic resistance independent of changes in their proliferative capacity, albeit subject to ruin‐mediated suppression.

Rapamycin, a well‐established therapeutic compound that has prominent senomorphic potential, can reduce the SASP expression without affecting viability of senescent cells (Deryabin & Borodkina, 2022; Xu, Shen, et al., 2021). We found that the inhibitory effects of rutin on cancer cell malignancy through restraining the cancer‐promoting capacity of stromal cell CM were generally reproduced by rapamycin, suggesting a common feature shared by these two agents (Figure S4c–f). Furthermore, to confirm the data with an alternative cancer type, we employed the primary human breast stromal cell line HBF1203, together with an established breast cancer (BCa) line, as exemplified by each of the triple negative breast cancer (TNBC) lines MDA‐MB‐231 and SUM159 (highly metastatic), or nonmetastatic lines T47D and MCF‐7, a technically mature coculture system we frequently employed (Chen et al., 2018; Zhang et al., 2018, 2021). The results largely phenocopied those we acquired from PCa assays, thus substantiating the senomorphic role of rutin in downregulating the protumorigenic activities of senescent cells (Figure S5a–e). Hence, targeting the full spectrum of SASP with a senomorphic agent like rutin, can significantly deprive cancer cells of stroma‐conferred gain‐of‐functions, particularly resistance to chemotherapeutic drugs, providing a baseline for development of new therapeutic regimens to improve anticancer outcomes.

### 2.5 Combination of chemotherapy with rutin improves treatment efficacy in preclinical trials

Given the effects of rutin on the expression profile and in vitro phenotypes of cancer cells, we next queried the therapeutic effectiveness that rutin may exhibit under in vivo conditions. To address this, we generated tissue recombinants by admixing PSC27 sublines with PC3 cells at a preoptimized ratio (1:4 in this study) before subcutaneous implantation to the hind flank of experimental mice with severe combined immunodeficiency (SCID). Animals were measured for tumor size at the end of an 8‐week period. Compared with tumors comprising PC3 and PSC27^Naive^, xenografts composed of PC3 and PSC27^SEN^ exhibited significantly enlarged sizes (Figure S6a). Nevertheless, pretreatment of PSC27^SEN^ cells with rutin in vitro prior to generation of tissue recombinants resulted in considerably reduced tumor volumes (*p* < 0.0001), largely reproducing the effectiveness of rapamycin, which can generate a long term in vivo consequence even after used to treat senescent human cells for once in vitro (Laberge et al., 2015).

**FIGURE 5:**
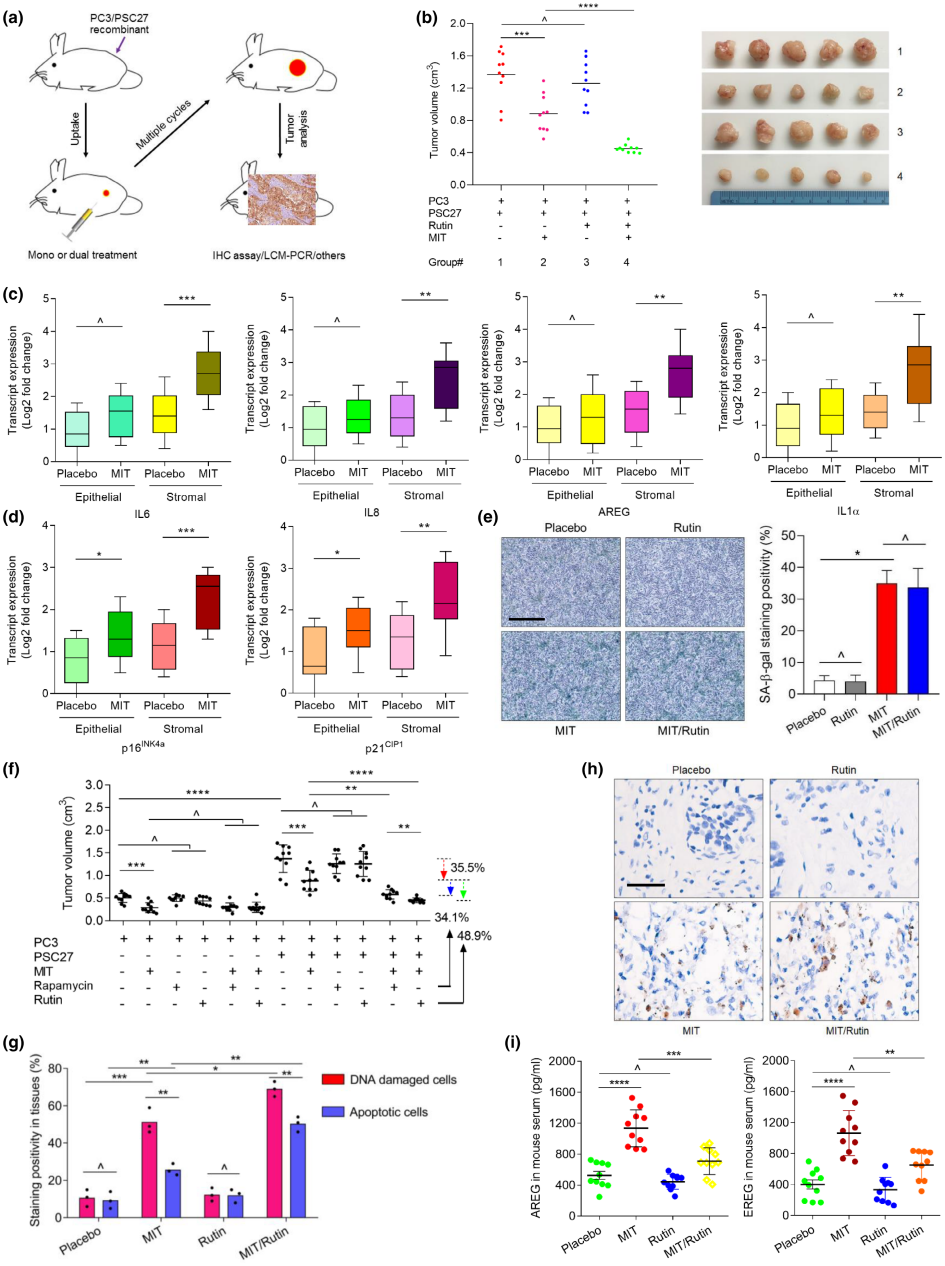
Combinational treatment of chemotherapy and rutin improves therapeutic outcome in preclinical trials. (a) A schematic workflow of experimental procedure for severe combined immunodeficient (SCID) mice. Two weeks after subcutaneous implantation and in vivo uptake of tissue recombinants, animals received either single or combinational agents administered as metronomic treatments composed of several cycles. (b) Statistical comparison of tumor end volumes between treatment groups. PC3 cells were xenograted alone or together with PSC27 cells to the hind flank of SCID mice. MIT was administered to induce tumor regression. Left, comparative statistic. Right, representative tumor images. (c) Expression assays by transcript level quantification of several canonical SASP factors expressed in stromal cells isolated from the tumors of SCID mice. Tissues from animals implanted with both stromal and cancer cells were subject to laser capture microdissection (LCM) isolation, total RNA preparation and expression assays. (d) Expression assessment by transcript level quantification of senescence biomarkers including p16^INK4a^ and p21^CIP1^. Tissues from animals were processed as described in (c). (e) Determination of cellular senescence in mouse tissues. Left, representative images of SA‐β‐Gal staining in xenografts of each group. Scale bar, 200 μm. Right, comparative statistics. (f) Statistical profiling of tumor growth in animals subject to several different treatment modalities. Mice were implanted with PC3 alone or in combination with PSC27, before treated by the chemotherapeutic drug (MIT) or combinational agents (MIT/Rutin). Tumor volumes were measured at the end of an 8‐week preclinical regimen. (g) Statistical assessment of DNA‐damaged and apoptotic cells in the tumor specimens analyzed in (e). Values are presented as percentage of cells positively stained by IHC with antibodies against γH2AX or caspase 3 (cleaved). (h) Representative IHC images of caspase 3 (cleaved) in tumors at the end of therapeutic regimens. Biopsies of placebo‐treated animals served as negative controls for MIT‐treated mice. Scale bar, 60 μm. (i) Assessment of circulation concentrations of two representative SASP factors (AREG and EREG) in the serum of experimental mice treated by chemotherapy and/or rutin. Data were derived from target‐specific ELISA assays. Left, AREG. Right, EREG. Data in c–i are representative of three independent experiments. Animal studies were performed with 10 mice *per* group (*n* = 10). All *p* values were calculated by Student's *t* tests. ^*p* > 0.05. *p < 0.05. **p < 0.01. ***p < 0.001. ****p < 0.0001.

To closely simulate clinical conditions that involve chemotherapy, we designed a preclinical regimen which incorporates genotoxic drugs and/or rutin (Figure 5a and Figure S6b). Two weeks post human cell implantation when stable uptake of tumors by host animals was generally observed, a single dose of MIT or placebo was administered at the 1st day of 3rd, 5th and, 7th week until the end of the 8‐week regimen (Figure S6b). In contrast to placebo, MIT treatment resulted in remarkably reduced tumor sizes, validating the efficacy of MIT as a cytotoxic agent (Figure 5b). Of note, there was a significant upregulation of SASP factors including IL6, IL8, AREG, IL1α, MMP1, MMP3, ANGPTL4, and SPINK1, concurrent with the induction of typical senescence markers including p16^INK4a^, p21^CIP1^, and SA‐β‐Gal in xenografts composed of PC3/PSC27 cells, implying development of an in vivo senescence and the SASP in response to MIT treatment (Figure 5c,d and Figure S6c). Interestingly, expression of certain SASP factors such as MMP3, together with the canonical senescence markers including p16^INK4A^ and p21^CIP1^, was induced by MIT in both stromal and cancer cell populations, suggesting chemotherapy caused comprehensive in vivo senescence, although the SASP profile seemed to be differently developed between these two cell subpopulations (Figure 5d and Figure S6c). SA‐β‐Gal staining results confirmed considerable tissue senescence induced by MIT, a case in sharp contrast to rutin, which seemed to neither promote nor suppress senescence (Figure 5e), a feature largely consistent with our in vitro observations (Figure 2a).

We next interrogated whether technically inhibiting development of the full SASP of treatment‐damaged stoma could further enhance the therapeutic response of tumors. To this end, we administered rapamycin, one of the well‐established senomorphic agents with excellent performance in targeting the SASP (Laberge et al., 2015), or rutin, with the chemotherapeutic drug MIT since the first dose of preclinical treatment. Though MIT per se caused shrinkage of PC3‐only tumors (*p* < 0.001), delivery of senomorphics did not show a remarkable effect (*p* > 0.05) (Figure 5f). It is noteworthy that these agents did not confer further benefits even when they were combined with MIT (*p* > 0.05), implying the independence of PC3 tumor growth on the tissue‐level SASP, specifically in the absence of stromal cells. Strikingly, upon combination of PC3 cells together with their stromal counterparts, we observed markedly increased tumor volumes (*p* < 0.0001), further validating the tumor‐promotive effect of stromal cells in vivo (Figure 5f). However, when animals harboring PC3/PSC27 tumors were exposed to MIT, tumor volumes remarkably decreased (35.5%, *p* < 0.001). Upon coadministration of either rapamycin or rutin with MIT as dual treatments, tumors displayed further shrinkage (34.1%, *p* < 0.01 and 48.9%, *p* < 0.0001, respectively) (Figure 5f).

To unmask the mechanism(s) inherently responsible for the SASP‐induced cancer resistance, we chose to dissect tumors from animals 7 days after initiation of treatment, a timepoint prior to development of resistant colonies. In contrast to placebo, MIT per se caused significant DNA damage and apoptosis in cancer cells (Figure 5g). Rutin alone caused neither a typical DDR nor enhanced cell death in PC3/PSC27 xenografts, suggesting limited responses of these tumors when animals were exposed to rutin only (Figure 5g). Upon combination with MIT, rutin further increased the percentage of cell apoptosis, implying an augmented cytotoxicity upon coadministration of MIT and rutin. The pattern of in vivo apoptosis was generally consistent with that of tumor regression upon treatment by different agents. Immunohistochemistry (IHC) staining indicated enhanced caspase 3 cleavage, a typical cell apoptosis indicator, when rutin was administered with MIT (Figure 5h). ELISA data suggested that MIT‐mediated chemotherapy resulted in elevated levels of circulating AREG and EREG in animals, a pattern that was basically reversed when rutin was delivered (Figure 5i).

Given the pronounced efficacy of combinational treatment in cancer therapy, we further expanded the study to confirm PCa results with breast tumors via generation of xenografts comprising MDA‐MB‐231 (malignant) and HBF1203 (stromal) cells, a combination that mimicks breast tumorigenesis as forementioned. Again, MDA‐MB‐231/HBF1203 tumors largely reproduced the results of preclinical experiments performed with PCa system (Figure S6d,e). Moreover, replacement of rutin with rapamycin, an alternative senomorphic agent, basically phenocopied those rutin‐involving preclinical assays (Figure S6f). The findings suggest that the resistance‐minimizing effects of the SASP‐targeting strategy with a senomorphic agent are not limited to a specific cancer type, but likely applicable to a wide range of solid malignancies.

To establish the safety and feasibility of such a therapeutic regimen, we performed a routine pathophysiological appraisal. The data supported that either single or combinational treatment was well tolerated, as evidenced by body weight maintenance throughout the therapeutic time frame (Figure S6g). No significant perturbations in serum levels of creatinine, urea, and metabolic activities of liver enzymes (ALP and ALT) were observed (Figure S6h). Additional data from treatment by MIT/rutin in immunocompetent animals (C57BL/6J background) also exhibited no changes of body weight, organ function, or routine blood counts, thus further validating the findings (Figure S7a–c). Together, these results evidently support that combining a senomorphic agent with conventional chemotherapy holds the prominent potential to enhance tumor response without causing severe cytotoxicities.

## 3 DISCUSSION

**FIGURE 6:**
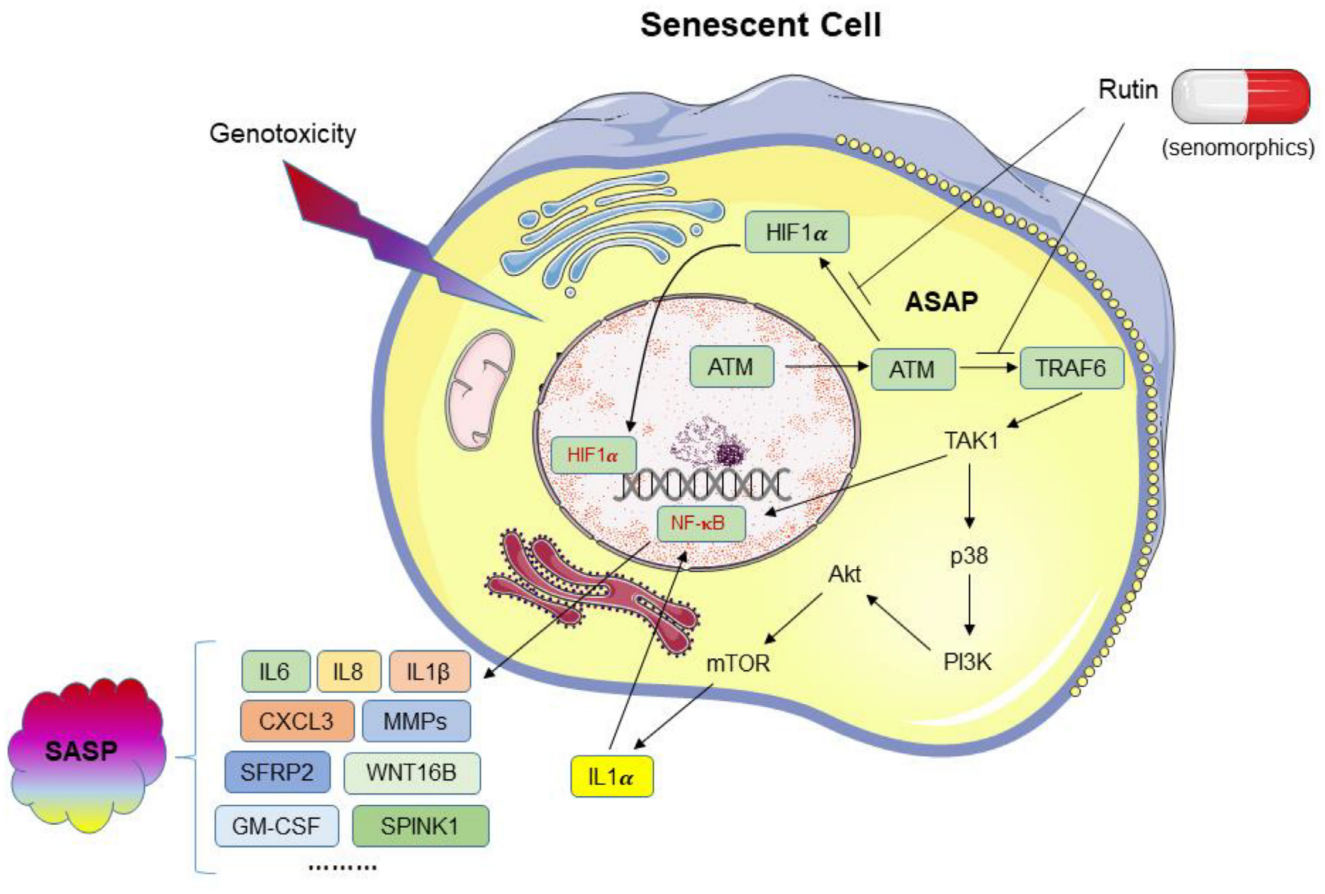
An illustrative working model of cellular senescence induction in response to environmental stimuli and the functional mechanism of rutin as a novel senomorphic agent. In the early stage of the SASP development (more specifically, during the ASAP phase), there are intermolecular interactions, including those between ATM and each of its two direct targets, namely HIF1α and TRAF6. As a senomorphic agent, rutin targets the interaction between ATM and HIF1α, resulting in reduced HIF1α activity and weakened downstream activities particularly those involved in regulating the SASP expression. At the same time, rutin‐mediated abrogation of the interaction between ATM and the other target, TRAF6, significantly retards signal transmission to its downstream molecules such as TAK1, which functionally connects the acute response ASAP, and the chronic development, usually termed the SASP.

Aging is a predominant risk factor for the vast majority of age‐related pathologies, raising the possibility that interventions into the basic aging process hold a substantial advantage for ameliorating many age‐related conditions. Aging research explores the functional decline of organisms during adulthood, while the interdependence of 12 aging hallmarks implies that changes into one specific hallmark may affect others as well (Lopez‐Otin et al., 2022). It is increasingly recognized that aging is a complex process, which indeed has to be conceived as a whole, with each of the hallmarks to be considered as a point‐of‐entry for exploration of the aging process and development of novel antiaging medicines. As a special contributor to the aging process, senescent cells accumulate with age in mammals and display a pro‐inflammatory SASP, thus can drive or exacerbate age‐related pathologies, including cancer. In this study, we screened a NMA library and discovered a novel senomorphic agent rutin, which is originally derived from natural plants, targets senescence‐associated pathways and efficiently represses the SASP, but without causing substantial adverse effects. The prominent senomorphic capacity of rutin supports its substantial value in future medicinal pipelines, particularly those correlated with geriatric medicine or antiaging health care (Figure 6).

Senescent cells are heterogeneous regarding their transcriptional landscapes, metabolic activities, and SASP profiles between species, cell types, and even individual cells, resulting in differential reliance on certain senescence‐mediating and prosurvival pathways. Targeting shared signaling pathways between senescent and proliferating cells can result in off‐target effects, posing a technical challenge to the safety and specificity of clinical interventions. For instance, ABT‐263 (navitoclax), a BCL‐2 family inhibitor, is not only senolytic to senescent cells, but also cytotoxic against platelets and neutrophils, often resulting in thrombocytopenia and neutropenia (Leverson et al., 2015). One of the central questions in geriatric medicine is whether the induction of senolysis of all types of senescent cells is truly necessary and represents the best therapeutic option. Apart from the issue of off‐target effects caused by senolytic targeting of critical pathways between senescent and proliferating or quiescent cells, senescent cells per se play essential roles in tissue repair, wound healing, embryonic development, and tumor prevention (Sun et al., 2018). In such a scenario, first of all, it is important that senotherapeutics still allow beneficial senescent cell populations to persist in the tissue microenvironment. Secondly, as senescent cells accumulate slowly overtime, senolytics are ideally to be administered periodically in metronomic regimens, thus may prevent radical eradication or overinterference of critical senescent cell‐driven processes (Zhang et al., 2022).

Given the pros and cons of senescent cells, senomorphics, which attenuate the pathological and pro‐inflammatory SASP without inducing cell senolysis, may indeed represent a better therapeutic approach. Since senomorphics still target senescence‐associated pathways, it is important that these molecules do not cause substantial adverse effects. Attenuating certain senescence phenotypes for a prolonged time may invoke global tissue dysregulation, and may suppress the generation of senescent cells in a number of physiological settings, such as prevention of carcinogenesis and dysregulation of tissue renewal. Of note, senomorphics, like senolytics, have been demonstrated to extend health span and prolong life span in experimental animals (Lagoumtzi & Chondrogianni, 2021). In our study, rutin displays a senomorphic, rather than senolytic activity in vitro, when used at an optimal concentration of 100 μM. In contrast to rapamycin, which though alleviates age‐related dysfunctions in model organisms, induces hyperlipidaemia, thrombocytopenia, metabolic dysregulation, and impairs wound healing (similar to navitoclax), rutin seems to be a moderate senomorphic agent with decent safety and limited cytotoxicity as evidenced by our preclinical studies. Thus, rutin can be considered as a practical senomorphic agent to be administered in a metronomic manner, such as biweekly or for even longer intervals as necessary, to suppress the pro‐inflammatory activity of slowly growing population of senescent cells.

Our work revealed the functional role of rutin as a novel senomorphic agent in targeting senescent cells, and disclosed the involvement of rutin in critical events in the early stage of the SASP development (or more specifically, during the ASAP), interaction of ATM with two of its direct targets, HIF1α and TRAF6. First, rutin suppresses the interaction between ATM and HIF1α, resulting in reduced HIF1α activity and weakened downstream effects particularly those on regulating the SASP expression. As a master regulator of cellular and systemic homeostasis activated in response to various inherent or environmental stimuli, HIF‐1α plays crucial role in facilitating damaged cells to sustain survival, while HIF‐1α deficiency increases cell apoptosis and reduces their surviving capacity (Hamidi et al., 2022). Upregulation of HIF‐1α occurs coincidently with engagement of the NF‐κB complex, while both activities are downstream events of STING signaling, which supports the lysosomal clearance of damaged DNA in response to oxidative stress (Chen et al., 2022). By contrast, HIF‐1α knockout cells can display enhanced DNA damage as demonstrated by increased generation and persistence of γH2AX foci, showing delayed DNA repair after genotoxic stresses such as ionizing radiation, indicative of affected nonhomologous end joining (NHEJ) repair capacity (Hamidi et al., 2022). Blockade of the interaction between ATM and HIF1α results in compromised signaling to allow activation of genes involved in cellular response to hypoxia, a condition comprehensively arising in senescent cells which develop mitochondrial dysfunction and generate reactive oxygen species (ROS) (Wiley et al., 2016). Abrogation of the interaction between ATM and the other target, TRAF6, disturbs signal transmission to downstream molecules such as TAK1, which connects the acute response, namely ASAP, and the chronic development, usually referred to as the SASP (Zhang et al., 2018). Thus, the senomorphic agent rutin targets senescent cells by acting on an early reaction chain, which involves ATM‐mediated activation of both HIF1α and TRAF6, key factors indispensable for signaling cascade that eventually culminates in the expression of a full spectrum SASP.

Our preclinical studies demonstrated that rutin has an excellent efficacy in promoting chemotherapeutic outcomes, such as in the case of MIT treatment. While cancer cells also become senescent in response to various chemotherapeutic regimens, they do have the capacity to repopulate and expand continuously within tumor foci during and/or after anticancer treatments. Indeed, therapy‐induced tumor regression is rarely durable, and the repopulation of recovering cancer cells frequently contributes to disease relapse (Appiah et al., 2023; Duy et al., 2021). Even a small number of drug‐resistant colonies can result in tumor relapse after completion of anticancer regimens (Liu et al., 2022). Despite the beneficial effects of chemotherapy, cancer cells undergoing therapy‐induced senescence (TIS) often re‐emerge as more aggressive colonies and significantly contribute to recurrence, for which reason senolytics and/or senomorphics are increasingly proposed as adjuvant treatments to deplete senescent cells in vivo (Liu et al., 2022; Sun et al., 2022). Notably, our data indicated that cancer epithelial cells did not exhibit a typical SASP, although manifesting increased expression of senescence markers such as p16^INK4a^ and p21^CIP1^, suggesting a differential response of these cells to chemotherapy as compared with their stromal counterparts. In fact, this is largely consistent with the observations of our recent studies (Chen et al., 2018; Zhang et al., 2018, 2021). It is reasonable to speculate that such a phenomenon is at least partially correlated with the repopulation of cancer cells, which can be more aggressive and show significantly enhanced malignancy after they survive anticancer treatments.

As effective SASP inhibitors, senomorphic agents could be used as ideal alternatives to senolytics, as they potentially preserve the less SASP‐dependent proimmunogenic capacity of senescent cells, particularly tumor immunosurveillance in the context of cancer cell senescence (Schmitt et al., 2022). Our results indicated that neither rapamycin nor rutin per se was able to generate significant effects on the growth of tumors, at least evidenced by data from those developed from xenografts composed of PC3 or MDA‐MB‐231 cells. However, MIT and DOX caused significant shrinkage of PCa and BCa tumors, respectively, suggesting the anticancer capacity of such typical chemotherapeutic agents is generally higher than that of rapamycin and rutin (senomorphics) when used alone, especially in the experimental settings of our study. Although former studies reported the effectiveness of these agents in cancer treatments (Ismail et al., 2023; Yin et al., 2023), the virtual efficacy of each of these senomorphics may be dependent on a number of specified factors such as the pharmacological dose, cancer type, and administration schedule of anticancer regimens.

Flavonoids are major dietary constituents of regular food prepared from the plant kingdom, while they are normally present in substantial amounts. Rutin is a flavonol‐type polyphenol which consists of the flavonol quercetin and the disaccharide rutinose (Budzynska et al., 2019). Importantly, rutin can exert diverse biological effects such as antitumor and antimicrobial actions, which are mainly associated to its antioxidant and anti‐inflammatory activities. To date, however, the complexity of natural rutin has impeded the development of rutin‐derived drugs, and detailed mechanism of rutin in human body after consumption remains largely unclear. Some studies focus on the development of extraction method to increase the extraction yield of rutin. As a natural flavonoid compound, rutin has displayed an extensive range of therapeutic potentials, while improving the bioavailability of rutin via novel drug delivery strategies may help optimize its medicinal use in more treatment settings. A recent study reported preparation of rutin nanomaterials for various therapeutic objects, suggesting enhanced aqueous solubility and elevated efficacy in contrast to conventional delivery approach, although further investigations are essential to confirm the improved bioavailability of rutin nanoformulations (Negahdari et al., 2021). Importantly, sodium rutin, a form of rutin artificial product, can extend the life span of mice by 10%, with sodium rutin supplementation holding the potential to alleviate age‐related pathological alterations and promote behavior performance in aged mice (Li et al., 2022). Together, the implications of rutin and its derived products in targeting senescent cells in these age‐related conditions merit further investigation, and future research outputs of such senomorphics will likely present a novel avenue to extend the life span and health span of humans by providing antiaging beneficial effects in geriatric medicine or even daily life.

## 4 METHODS

### 4.1 Cell culture

Primary normal human prostate stromal cell line PSC27 and breast stromal cell line HBF1203 were courteous gifts from Dr. Peter Nelson (Fred Hutchinson CRC) and maintained in stromal complete medium as described previously (Sun et al., 2012). Human fetal lung stromal lines WI38 and HFL1, and foreskin stromal line BJ were from ATCC and cultured with F‐12 K medium supplemented with 10% FBS. Prostate cancer epithelial cell lines PC3, DU145, and LNCaP, as well as breast cancer epithelial cell lines MDA‐MB‐231, SUM159, T47D, and MCF‐7 (ATCC) were routinely cultured with RPMI 1640 (10% FBS). Prostate cancer epithelial line M12 was kindly provided by Dr. Stephen Plymate (University of Washington), which originally derived from the benign line BPH1 but phenotypically neoplastic and metastatic (Bae et al., 1998). All lines were routinely tested for mycoplasma contamination and authenticated with STR assays.

### 4.2 Cell treatments

Stromal cells were grown until 80% confluent (CTRL) and treated with 50 μg/mL bleomycin (BLEO). After treatment, the cells were rinsed briefly with PBS and allowed to stay for 7–10 days prior to performance of various examinations. Senotherapeutic agents screened in this study were purchased from Shyuanye Biotechnology (http://www.shyuanye.com). To perform drug screening to search for potential senolytics, natural candidates (totally 37 in the NMA library) were tested each at 3 μg/mL on the survival of 5.0 × 10^3^ control and senescent cells for 3 d. Subsequently, appraisal of the effects of remaining agents (37 in the library, each applied at 1 μg/mL) was performed to test the efficacy of SASP inhibition (senomorphics). For those of outstanding potential to act as effective and safe senomorphics, further assessments were followed. The phytochemical agent rutin was applied in the range of 20 μM to 100 μM, with 100 μM determined to be an optimal concentration for the senomorphics purpose. The small molecule inhibitor PX‐478 was used at 10 μM, while C25‐140 was employed at 20 μM, to treat senescent cells in culture before collection for lysis and expression analysis. Alternatively, cells were subject to treatment with the HIF1ɑ agonist VH298 at 200 μM or the TRAF6 activator IL17A at 10 ng/mL (Liu et al., 2023; Qiu et al., 2019), before subsequent cell process and lysis for expression assessment.

### 4.3 HPLC‐QTOF‐MS/MS analysis

HPLC‐QTOF‐MS/MS (high performance liquid chromatograph conjugated with quadrupole time‐of‐flight tandem mass spectrometer) analysis was performed on Nexera X2 LC‐40 (SHIMADZU) coupled to AB SCIEX TripleTOF 6600 LC/MS/MS system (SCIEX), data processed with Analyst TF (v 1.7.1). Briefly, the chromatographic separation was carried out on an Accucore C30 column (2.6 um, 250 × 2.1 mm, Thermo) at room temperature. The mobile phase solvent comprises 1% acetic acid in water (solvent A) and 100% methanol (solvent B). The multistep linear gradient solvent system started with 5% B and increased to 15% B (5 min), 35% B (30 min), 70% B (45 min), 70% B (49 min), and 100% B (50 min), held at 100% B for 10 min, and decreasing to 5% B (60 min). At the initial and last gradient step, the column was equilibrated and maintained or washed for 10 min with 5% B. The flow rate was 0.2 mL/min, while the injection volume of samples was 10 μL. Detection was performed using a TripleTOF 6600 qTOF mass spectrometer (AB SCIEX) equipped with an ESI interface in negative ion mode. Operation conditions of the ESI source were as follows: capillary voltage, −4000 V; drying gas, 60 (arbitrary units); nebulization gas pressure, 60 psi; capillary temperature, 650°C; and collision energy, 30. The mass spectra were scanned from 50 to 1200 m/z, at an acquisition rate 3 spectra per second. Data acquisition and analysis were performed using Analyst TF (AB SCIEX, v 1.7.1) software.

### 4.4 Measurement of intracellular reactive oxygen species

Intracellular reactive oxygen species (ROS) levels were determined using a ROS Assay Kit (Beyotime) which employs dichloro‐dihydro‐fluorescein diacetate (DCFH‐DA) as a probe. Briefly, cells were cultured in 6‐well plates for 24 h at 37°C and were then washed twice with serum‐free medium. Medium containing 10 μM DCFH‐DA was added. Cells were then incubated for 20 min at 37°C, with light avoided during incubation. After incubation, the cells were washed thrice with serum‐free medium, then observed and photographed using a fluorescence microscope (Nikon). The fluorescence intensity was measured using ImageJ software (v1.51, NIH).

### 4.5 RNA‐seq and bioinformatics analysis

Total RNA samples were obtained from senescent PSC27 cells cultured with regular DMEM or DMEM containing rutin (100 μM) for three consecutive days. Sample quality was validated by Bioanalyzer 2100 (Agilent), and RNA was subjected to sequencing by Illumina NovaSeq 6000 with gene expression levels quantified by the software package RSEM (https://deweylab.github.io/RSEM/). Briefly, rRNAs in the RNA samples were eliminated using the RiboMinus Eukaryote kit (Qiagen, Valencia, CA, USA), and strand‐specific RNA‐seq libraries were constructed using the TruSeq Stranded Total RNA preparation kits (Illumina, San Diego, CA, USA) according to the manufacturer's instructions before deep sequencing.

Pair‐end transcriptomic reads were mapped to the reference genome (GRCh38.p14) (Genome Reference Consortium Human Build 38; INSDC Assembly GCA_000001405.28, 12.2013) (http://asia.ensembl.org/Homo_sapiens/Info/Index) (ensembl_105) with reference annotation from GENCODE v42 using the Bowtie tool. Duplicate reads were identified using the picard tools (1.98) script mark duplicates (https://github.com/broadinstitute/picard) and only nonduplicate reads were retained. Reference splice junctions are provided by a reference transcriptome (Ensembl build 73) (Zerbino et al., 2015). FPKM values were calculated using Cuff links, with differential gene expression called by the Cuffdiff maximum likelihood estimate function (Trapnell et al., 2012). Genes of significantly changed expression were defined by a false discovery rate (FDR)‐corrected *p* value <0.05. Only ensembl genes 73 of status “known” and biotype “coding” were used for downstream analysis.

Reads were trimmed using Trim Galore (v0.6.1) (http://www.bioinformatics.babraham.ac.uk/projects/trim_galore/) and quality assessed using FastQC (v0.10.0) (http://www.bioinformatics.bbsrc.ac.uk/projects/fastqc/). Differentially expressed genes were subsequently analyzed for enrichment of biological themes using the DAVID bioinformatics platform (https://david.ncifcrf.gov/), the Ingenuity Pathways Analysis program (http://www.ingenuity.com/index.html). Raw data of RNA‐seq were deposited in the NCBI Gene Expression Omnibus (GEO) database under the accession code GSE190279.

#### 4.5.1 Venn diagrams

Venn diagrams and associated empirical *p* values were generated using the USeq (v7.1.2) tool IntersectLists (Nix et al., 2008). The t value used was 22,008, as the total number of genes of status “known” and biotype “coding” in ensembl genes 73. The number of iterations used was 1,000.

#### 4.5.2 RNA‐seq heatmaps

For each gene, the FPKM value was calculated based on aligned reads, using Cuff links (Trapnell et al., 2012). Z‐scores were generated from FPKMs. Hierarchical clustering was performed using the R package heatmap.2 and the distfun = “pearson” and hclustfun = “average”.

### 4.6 Mapping of unique interactors with high‐throughput datasets

Analysis of ATM‐ and TRAF6‐interactive molecules was performed with BioGRID (v4.4.217), a biomedical interaction repository with data compiled through comprehensive curation efforts and used as a public database archiving and disseminating genetic and protein interaction data from model organisms and humans (thebiogrid.org) (Oughtred et al., 2019; Stark et al., 2006). BioGRID searches 81,599 publications for 2,579,588 protein and genetic interactions, 30,725 chemical interactions and 1,128,339 post‐translational modifications from major model organism species, with human selected as the target species throughout this study.

### 4.7 Immunoblot and immunofluorescence analysis

Whole cell lysates were prepared using RIPA lysis buffer supplemented with protease/phosphatase inhibitor cocktail (Biomake). Nitrocellulose membranes were incubated overnight at 4°C with primary antibodies, and HRP‐conjugated goat anti‐mouse or ‐rabbit served as secondary antibodies (Vazyme). For immunofluorescence analysis, cells were fixed with 4% formaldehyde and permeabilized before incubation with primary and secondary antibodies, each for 1 h. Upon counterstaining with DAPI (0.5 μg/mL), samples were examined with an Imager A2. Axio (Zeiss) upright microscope to analyze specific gene expression.

### 4.8 In vitro cell phenotypic characterization

For proliferation assays of cancer cells, 2 × 10^4^ cells were dispensed into 6‐well plates and cocultured with conditioned medium (CM) from stromal cells. Three days later, cells were digested and counted with hemacytometer. For migration assays, cells were added to the top chambers of transwells (8 μm pore), while stromal CM were given to the bottom. Migrating cells in the bottom chambers were stained by DAPI 12–24 h later, with samples examined with an Observer A1. Axio (Zeiss) inverted microscope. Invasion assays were performed similarly with migration experiments, except that transwells were coated with basement membrane matrix (phenol red free, Corning). Alternatively, cancer cells were subject to wound healing assays conducted with 6‐well plates, with healing patterns graphed with bright‐field microscope. For chemoresistance assays, cancer cells were incubated with stromal CM, with the chemotherapeutic agent MIT provided in wells for 3 days at each cell line's IC50, a value experimentally predetermined. Cell viability was assayed by a CCK‐8 kit, with the absorbance at 450 nm measured using a microplate reader.

### 4.9 Histology and immunohistochemistry

Mouse tissue specimens were fixed overnight in 10% neutral‐buffered formalin and processed for paraffin embedding. Standard staining with hematoxylin/eosin was performed on sections of 5–8 μm thickness cut from each specimen block. For immunohistochemistry, tissue sections were deparaffinized and incubated in citrate buffer at 95°C for 40 min for antigen retrieval before incubated with primary antibodies (e.g., anticleaved Caspase 3, 1:1000) overnight at 4°C. After 3 washes with PBS, tissue sections were incubated with biotinylated secondary antibody (1:200 dilution, Vector Laboratories) for 1 h at room temperature then washed thrice, after which streptavidin‐horseradish peroxidase conjugates (Vector Laboratories) were added and the slides incubated for 45 min. DAB solution (Vector Laboratories) was then added and the slides were counterstained with hematoxylin.

### 4.10 Experimental animals and chemotherapeutic studies

All animals were maintained in a specific pathogen‐free (SPF) facility, with NOD/SCID (Charles River and Nanjing Biomedical Research Institute of Nanjing University) mice at an age of approximately 6 weeks (~20 g body weight) used. Ten mice were incorporated in each group, and xenografts were subcutaneously generated at the hind flank upon anesthesia mediated by isoflurane inhalation. Stromal cells (PSC27 or HBF1203) were mixed with cancer cells (PC3 or MDA‐MB‐231) at a ratio of 1:4 (i.e., 250,000 stromal cells admixed with 1,000,000 cancer cells to make tissue recombinants before implantation in vivo). Animals were sacrificed at 2–8 weeks after tumor xenografting, according to tumor burden or experimental requirements. Tumor growth was monitored weekly, with tumor volume (v) measured and calculated according to the tumor length (l), width (w) and height (h) by the formula: v = (π/6) × ((l + w + h)/3) (Chen et al., 2018; Kirkland & Tchkonia, 2020). Freshly dissected tumors were either snap‐frozen or fixed to prepare FFPE samples. Resulting sections were used for IHC staining against specific antigens or subject to hematoxylin/eosin staining.

For chemoresistance studies, animals received subcutaneous implantation of tissue recombinants as described above and were given standard laboratory diets for 2 weeks to allow tumor uptake and growth initiation. Starting from the 3rd week (tumors reaching 4–8 mm in diameter), MIT (0.2 mg/kg doses), DOX (doxorubicin, 1.0 mg/kg doses), the senomorphic agent rutin (10.0 mg/kg doses, 200 μL/dose), or vehicle controls was administered by intraperitoneal injection (therapeutic agents via i.p. route), on the 1st day of 3rd, 5th, and 7th weeks, respectively. Upon completion of the 8‐week therapeutic regimen, animals were sacrificed, with tumor volumes recorded and tissues processed for histological evaluation. Alternatively, rapamycin (10.0 mg/kg doses, 200 μL/dose) was used to replace rutin for above assays, with a similar procedure followed.

At the end of chemotherapy and/or targeting treatment, animals were anaesthetized and peripheral blood was gathered via cardiac puncture. Blood was transferred into a 1.5 mL Eppendorf tube and kept on ice for 45 min, followed by centrifugation at 9000 ×*g* for 10 min at 4°C. Clear supernatants containing serum were collected and transferred into a sterile 1.5 mL Eppendorf tube. All serum markers were measured using dry‐slide technology on IDEXX VetTest 8008 chemistry analyzer (IDEXX). About 50 μL of the serum sample was loaded on the VetTest pipette tip before securely fit on the pipettor and manufacturer's instructions were followed for further examination.

All animal experiments were performed in compliance with NIH Guide for the Care and Use of Laboratory Animals (National Academies Press, 2011) and the ARRIVE guidelines, and were approved by the Institutional Animal Care and Use Committee (IACUC) of Shanghai Institute of Nutrition and Health, Chinese Academy of Sciences.

### 4.11 Tissue SA‐β‐Gal staining and histological examination

For SA‐β‐Gal staining, frozen sections were dried at 37 °C for 20–30 min before fixed for 15 min at room temperature. The frozen sections were washed thrice with PBS and incubated with SA‐β‐Gal staining reagent (Beyotime) overnight at 37 °C. After completion of SA‐β‐Gal staining, sections were stained with eosin for 1–2 min, rinsed under running water for 1 min, differentiated in 1% acid alcohol for 10–20 s, and washed again under running water for 1 min. Sections were dehydrated in increasing concentrations of alcohol and cleared in xylene. After drying, samples were examined under a bright‐field microscope.

### 4.12 In vivo cytotoxicity evaluation by blood tests

For routine blood examination, 100 μL fresh blood was acquired from each animal and mixed with EDTA immediately. The blood samples were analyzed with Celltac Alpha MEK‐6400 series hematology analyzers (Nihon Kohden). For serum biochemical analyses, blood samples were collected and clotted for 2 h at room temperature or overnight at 4°C. Samples were then centrifuged (1000 × *g*, 10 min) to obtain serum. An aliquot of approximately 50 μL serum was subject to analysis for creatinine, urea, alkaline phosphatase (ALP), and alanine transaminase (ALT) by an automatic biochemical analyzer (BS‐5800 M, Mindray Bio‐Medical Electronics Co. Ltd). Evaluation of circulating levels of hemoglobin, white blood cells, lymphocytes, and platelets were performed using dry‐slide technology on a VetTest 8008 chemistry analyzer (IDEXX) as reported previously (Chen et al., 2018).

All animal experiments were conducted in compliance with the NIH Guide for the Care and Use of Laboratory Animals (National Academies Press, 2011) and the ARRIVE guidelines, and were approved by the IACUC of Shanghai Institute of Nutrition and Health, Chinese Academy of Sciences. For each preclinical regimen, animals were monitored for conditions including hypersensitivity (changes in body temperature, altered breathing, and ruffled fur), body weight, mortality, and changes in behavior (i.e., loss of appetite and distress), and were disposed of appropriately according to the individual pathological severity as defined by relevant guidelines.

### 4.13 Statistical analysis

All in vitro experiments were performed in triplicates, while animal studies were conducted with at least 10 mice *per* group for most preclinical assays. Data are presented as mean ± SD except where otherwise indicated. GraphPad Prism 8.4.3 was used to collect and analyze data, with statistical significance determined according to individual settings. Cox proportional hazards regression model and multivariate Cox proportional hazards model analysis were performed with statistical software SPSS. Statistical significance was determined by unpaired two‐tailed Student's *t* test, one‐ or two‐way ANOVA, Pearson's correlation coefficients test, Kruskal–Wallis, log‐rank test, Wilcoxon–Mann–Whitney test, or Fisher's exact test. For all statistical tests, a *p* value <0.05 was considered significant.

## AUTHOR CONTRIBUTIONS

Y.S. conceived this study, designed the experiments, and orchestrated the project. H.L. performed most of the in vitro assays and part of the in vivo experiments. Q.X. provided datasets from primary screening of drug library and key suggestions for biological experiments. Z.L. performed HPLC‐MS analysis of selected senomorphics. H.W. helped with graphic drawing of the chemical formula of rutin. R.S. and X.D. performed some cell culture and drug treatment assays. Q.F. performed partial preclinical studies and provided animal data. J.C. provided critical conceptual inputs. Y.S. performed data analysis, graphic presentation, and finalized the manuscript. All authors critically read and commented on the final manuscript.

## CONFLICT OF INTEREST STATEMENT

The authors declare no competing interests.

## Supporting information


Appendix S1

## Data Availability

Data supporting the plots within this paper and other findings of this study are available from the corresponding author upon reasonable request. The RNA‐seq data generated in the present study have been deposited in the NCBI Gene Expression Omnibus (GEO) database under accession code GSE190279.
