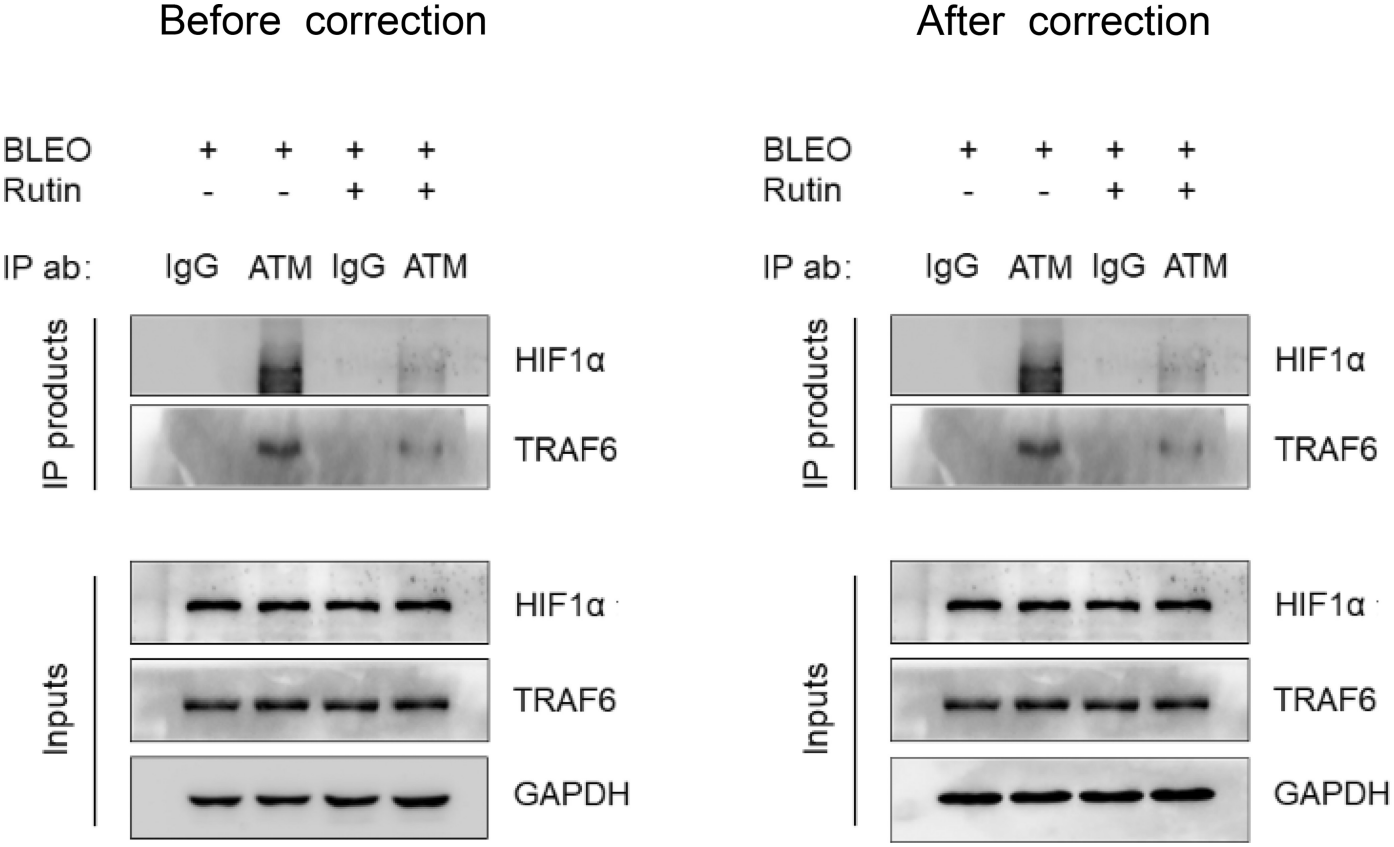# Correction to “Rutin is a potent senomorphic agent to target senescent cells and can improve chemotherapeutic efficacy”

**DOI:** 10.1111/acel.14183

**Published:** 2024-05-12

Liu, H., Xu, Q., Wufuer, H., Li, Z., Sun, R., Jiang, Z., Dou, X., Fu, Q., Campisi, J., Sun, Y. 2024. Rutin is a potent senomorphic agent to target senescent cells and can improve chemotherapeutic efficacy. *Aging Cell* 23(1): e13921; https://doi.org/10.1111/acel.13921

During the data organization and author preparation of this manuscript, there was an error inadvertently incorporated into the manuscript and not recognized effectively during the proofing stage. We noticed that the following item needs to be appropriately corrected.

1. Figure 3c. For assay of IP followed by immunoblot assessment with the whole lysates of stromal cells, the GAPDH piece was mistakenly picked up by authors to organize the original panel. As a necessary effort, the authors have now corrected this figure. Please refer to the updated Figure 3c.

All other parts of this article remain intact, valid, and unchanged. The authors sincerely regret the error and would like to apologize for any inconvenience this may have caused. The corrected figure is provided below.