# Correction to “Rutin is a Potent Senomorphic Agent to Target Senescent Cells and Can Improve Chemotherapeutic Efficacy”

**DOI:** 10.1111/acel.14488

**Published:** 2025-01-17

Liu, H., Xu, Q., Wufuer, H., Li, Z., Sun, R., Jiang, Z., Dou, X., Fu, Q., Campisi, J., Sun, Y. (2024). Rutin is a potent senomorphic agent to target senescent cells and can improve chemotherapeutic efficacy. *Aging Cell* 23(1): e13921.

In Figure 5e, for SA‐β‐Gal staining of mouse tissues, the Placebo image was mistakenly picked up to make the original panel. The corrected figure is provided below.

**Before Correction**

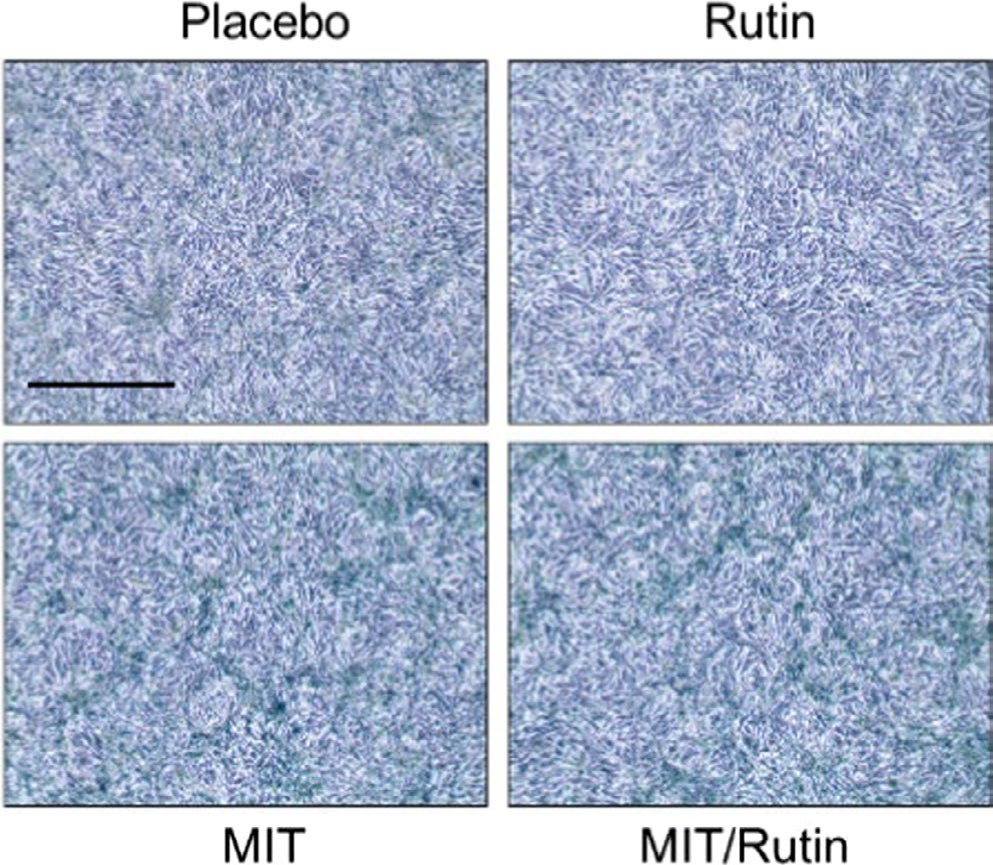

**After Correction**

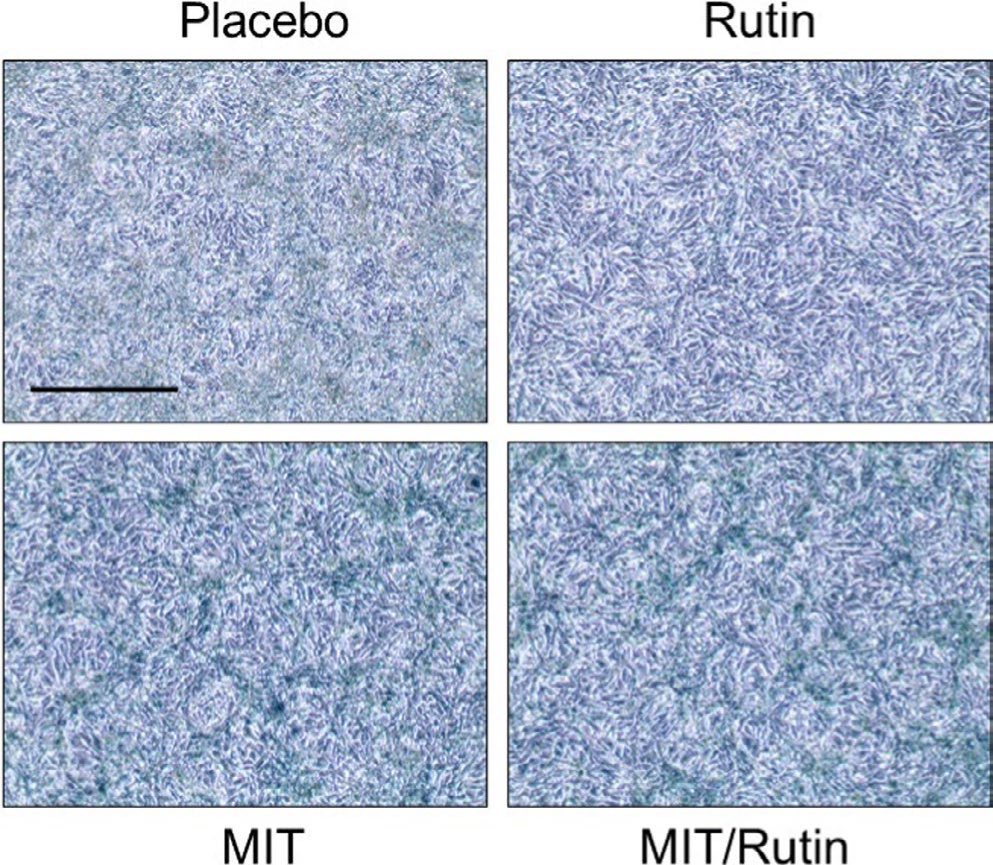

We apologize for this error.

