# Supplementary material for: Rutin is a potent senomorphic agent to target senescent cells and can improve chemotherapeutic efficacy
Source: Aging Cell. 2023 Jul 20;23(1):e13921. doi: 10.1111/acel.13921 (PMC10776113; doi:10.1111/acel.13921)
Supplement: Supplementary file 1 — Appendix S1 [file ACEL-23-e13921-s001.pdf]

Figure S1

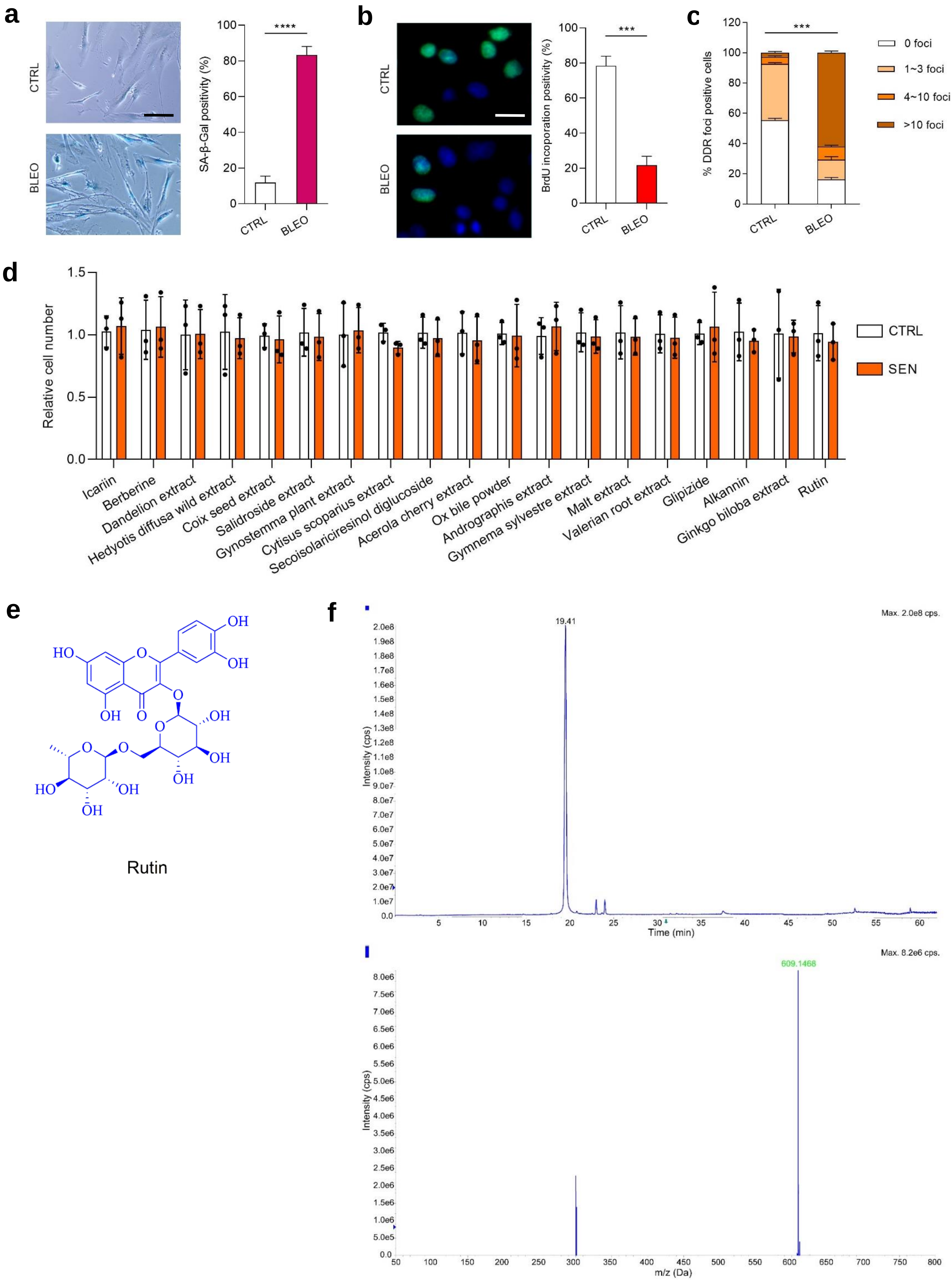
a
CTRL
BLEO
SA-β-Gal positivity (%)
****
b
BrdU incoporation positivity (%)
***
c
% DDR foci positive cells
0 foci
1~3 foci
4~10 foci
>10 foci
d
Relative cell number
CTRL
SEN
Icariin
Berberine
Dandelion extract
Hedyotis diffusa wild extract
Coix seed extract
Salidroside extract
Gynostemma plant extract
Cytisus scoparius extract
Secoisolariciresinol diglucoside
Acerola cherry extract
Ox bile powder
Andrographis extract
Gymnema sylvestre extract
Malt extract
Valerian root extract
Glipizide
Alkannin
Ginkgo biloba extract
Rutin
e
Rutin
f
Max. 2.0e8 cps.
19.41
Intensity (cps)
Time (min)
Max. 8.2e6 cps.
609.1468
m/z (Da)

Figure S2

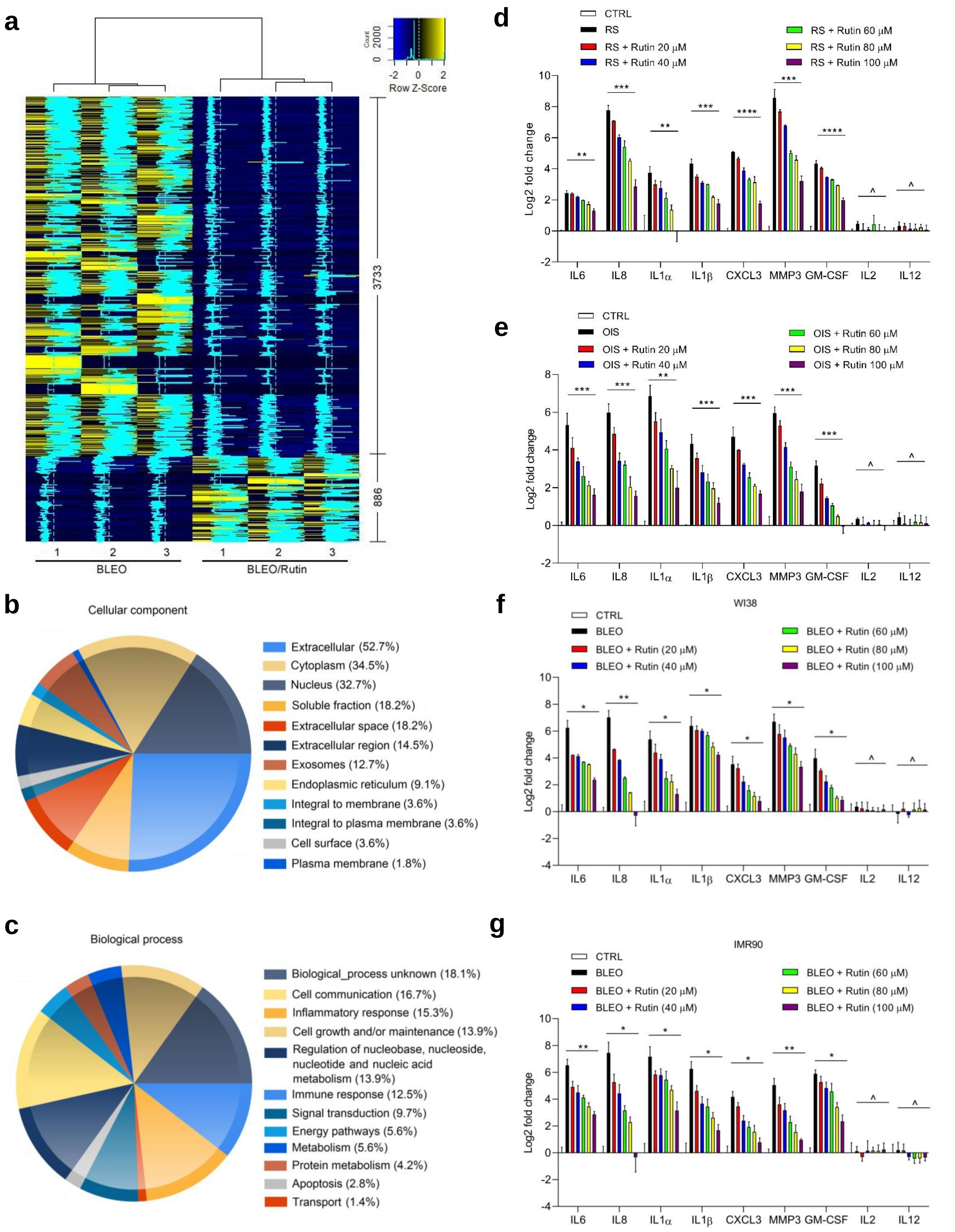

a
Count
Row Z-Score
3733
886
1 2 3 1 2 3
BLEO
BLEO/Rutin
b
Cellular component
Extracellular (52.7%)
Cytoplasm (34.5%)
Nucleus (32.7%)
Soluble fraction (18.2%)
Extracellular space (18.2%)
Extracellular region (14.5%)
Exosomes (12.7%)
Endoplasmic reticulum (9.1%)
Integral to membrane (3.6%)
Integral to plasma membrane (3.6%)
Cell surface (3.6%)
Plasma membrane (1.8%)
c
Biological process
Biological_process unknown (18.1%)
Cell communication (16.7%)
Inflammatory response (15.3%)
Cell growth and/or maintenance (13.9%)
Regulation of nucleobase, nucleoside, nucleotide and nucleic acid metabolism (13.9%)
Immune response (12.5%)
Signal transduction (9.7%)
Energy pathways (5.6%)
Metabolism (5.6%)
Protein metabolism (4.2%)
Apoptosis (2.8%)
Transport (1.4%)
d
CTRL
RS
RS + Rutin 20 μM
RS + Rutin 40 μM
RS + Rutin 60 μM
RS + Rutin 80 μM
RS + Rutin 100 μM
Log2 fold change
IL6 IL8 IL1α IL1β CXCL3 MMP3 GM-CSF IL2 IL12
e
CTRL
OIS
OIS + Rutin 20 μM
OIS + Rutin 40 μM
OIS + Rutin 60 μM
OIS + Rutin 80 μM
OIS + Rutin 100 μM
Log2 fold change
IL6 IL8 IL1α IL1β CXCL3 MMP3 GM-CSF IL2 IL12
f
WI38
CTRL
BLEO
BLEO + Rutin (20 μM)
BLEO + Rutin (40 μM)
BLEO + Rutin (60 μM)
BLEO + Rutin (80 μM)
BLEO + Rutin (100 μM)
Log2 fold change
IL6 IL8 IL1α IL1β CXCL3 MMP3 GM-CSF IL2 IL12
g
IMR90
CTRL
BLEO
BLEO + Rutin (20 μM)
BLEO + Rutin (40 μM)
BLEO + Rutin (60 μM)
BLEO + Rutin (80 μM)
BLEO + Rutin (100 μM)
Log2 fold change
IL6 IL8 IL1α IL1β CXCL3 MMP3 GM-CSF IL2 IL12

Figure S3

a

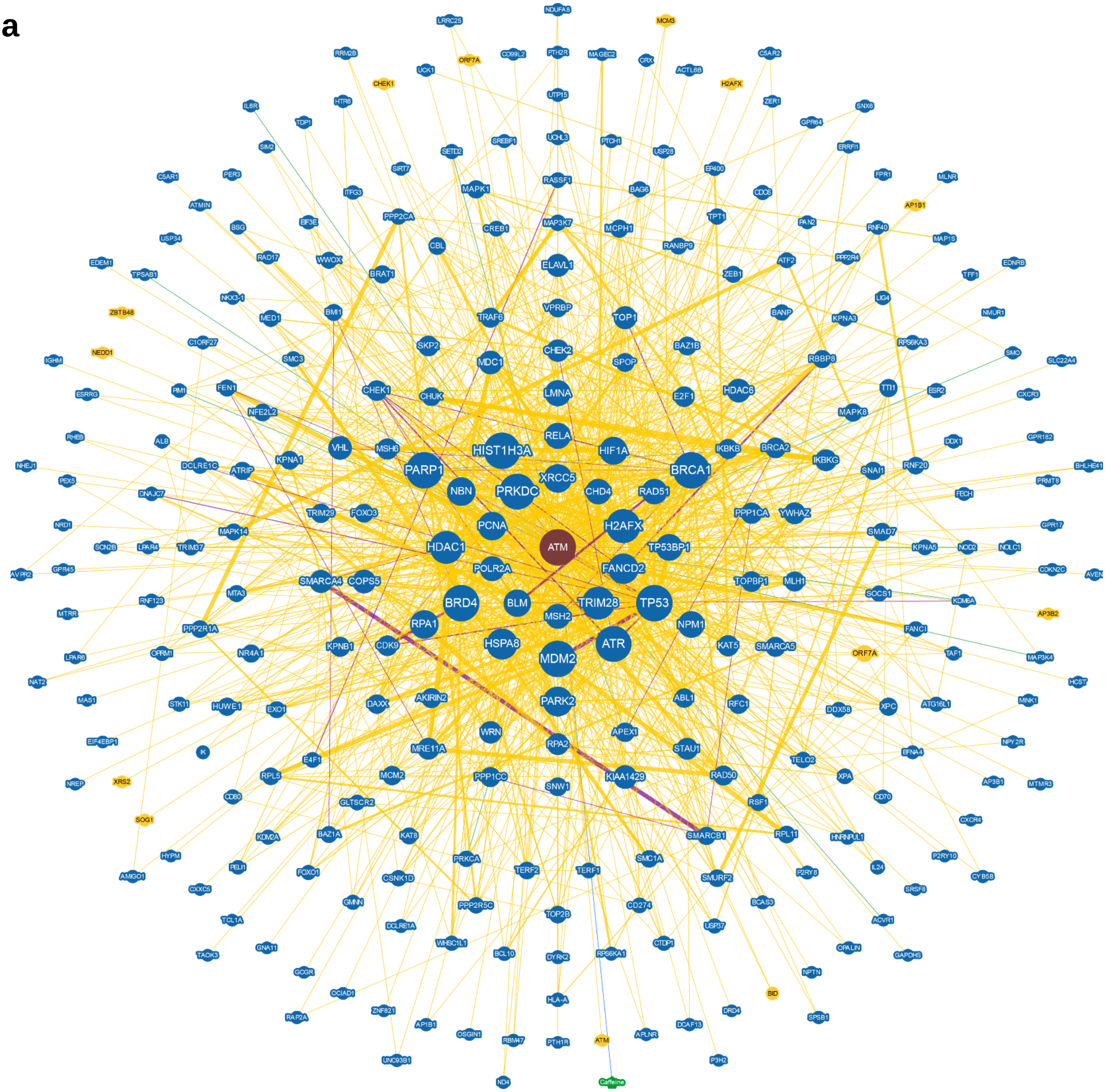

Figure S3

b

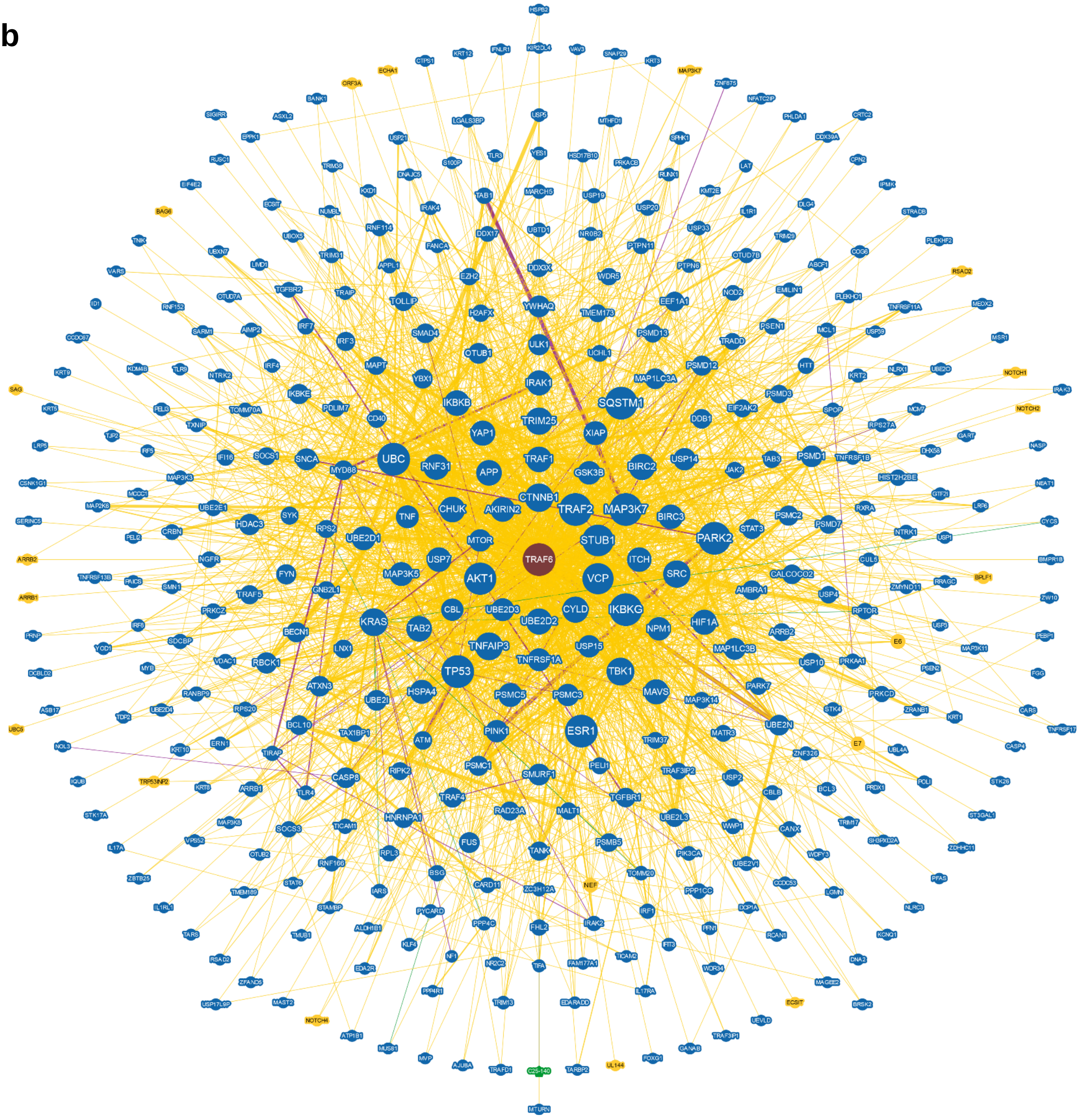

TRAF6
TRAF2
MAP3K7
SQSTM1
UBC
IKBKG
ESR1
TP53
KRAS
AKT1
VCP
STUB1
PARK2
CTNNB1
TRAF1
TRIM25
IRAK1
IKBKB
YAP1
APP
RNF31
GSK3B
BIRC2
XIAP
USP14
PSMD1
BIRC3
SRC
ITCH
MTOR
USP7
CHUK
AKIRIN2
TNF
UBE2D1
MAP3K5
CBL
UBE2D3
UBE2D2
CYLD
NPM1
HIF1A
TAB2
TNFAIP3
TNFRSF1A
USP15
TBK1
MAVS
HSPA4
PSMC5
PSMC3
PINK1
SNCA
MYD88
HDAC3
FYN
SYK
BECN1
RBCK1
ATXN3
UBE2N
USP10
CASP8
SMURF1
TRAF4
HNRNPA1
FUS

c

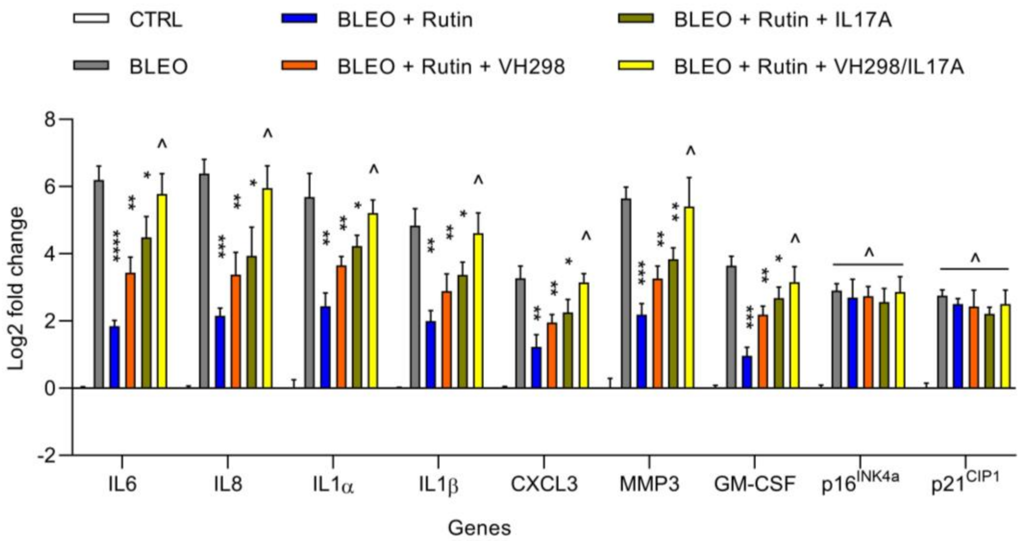

CTRL
BLEO
BLEO + Rutin
BLEO + Rutin + VH298
BLEO + Rutin + IL17A
BLEO + Rutin + VH298/IL17A
Log2 fold change
8
6
4
2
0
-2
IL6
IL8
IL1α
IL1β
CXCL3
MMP3
GM-CSF
p16INK4a
p21CIP1
Genes

Figure S4

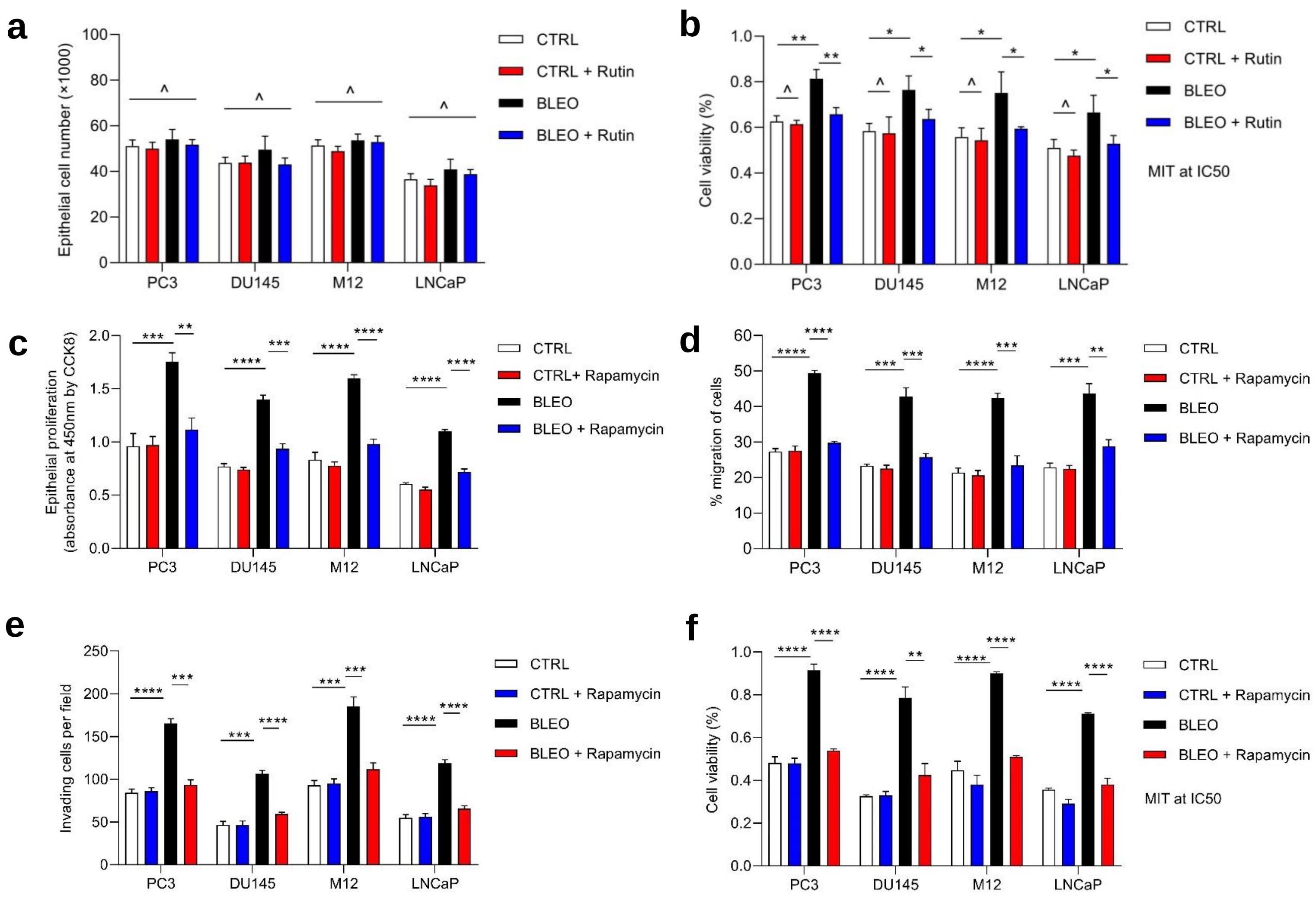
a
Epithelial cell number (×1000)
PC3
DU145
M12
LNCaP
CTRL
CTRL + Rutin
BLEO
BLEO + Rutin
b
Cell viability (%)
MIT at IC50
c
Epithelial proliferation (absorbance at 450nm by CCK8)
CTRL+ Rapamycin
BLEO + Rapamycin
d
% migration of cells
CTRL + Rapamycin
e
Invading cells per field
f
Cell viability (%)
MIT at IC50

Figure S5

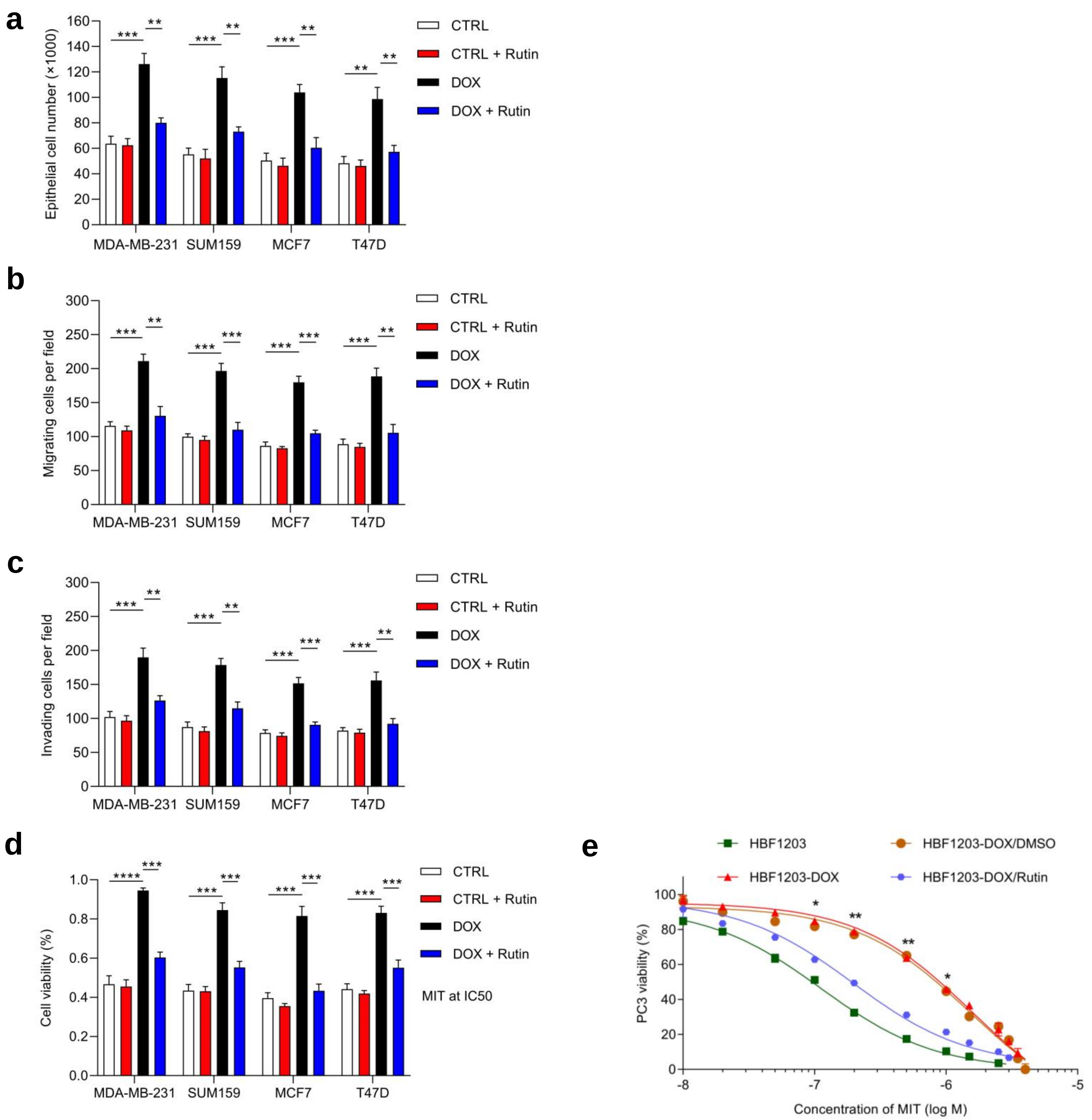
a
Epithelial cell number (×1000)
CTRL
CTRL + Rutin
DOX
DOX + Rutin
MDA-MB-231
SUM159
MCF7
T47D
b
Migrating cells per field
c
Invading cells per field
d
Cell viability (%)
MIT at IC50
e
HBF1203
HBF1203-DOX
HBF1203-DOX/DMSO
HBF1203-DOX/Rutin
PC3 viability (%)
Concentration of MIT (log M)

Figure S6

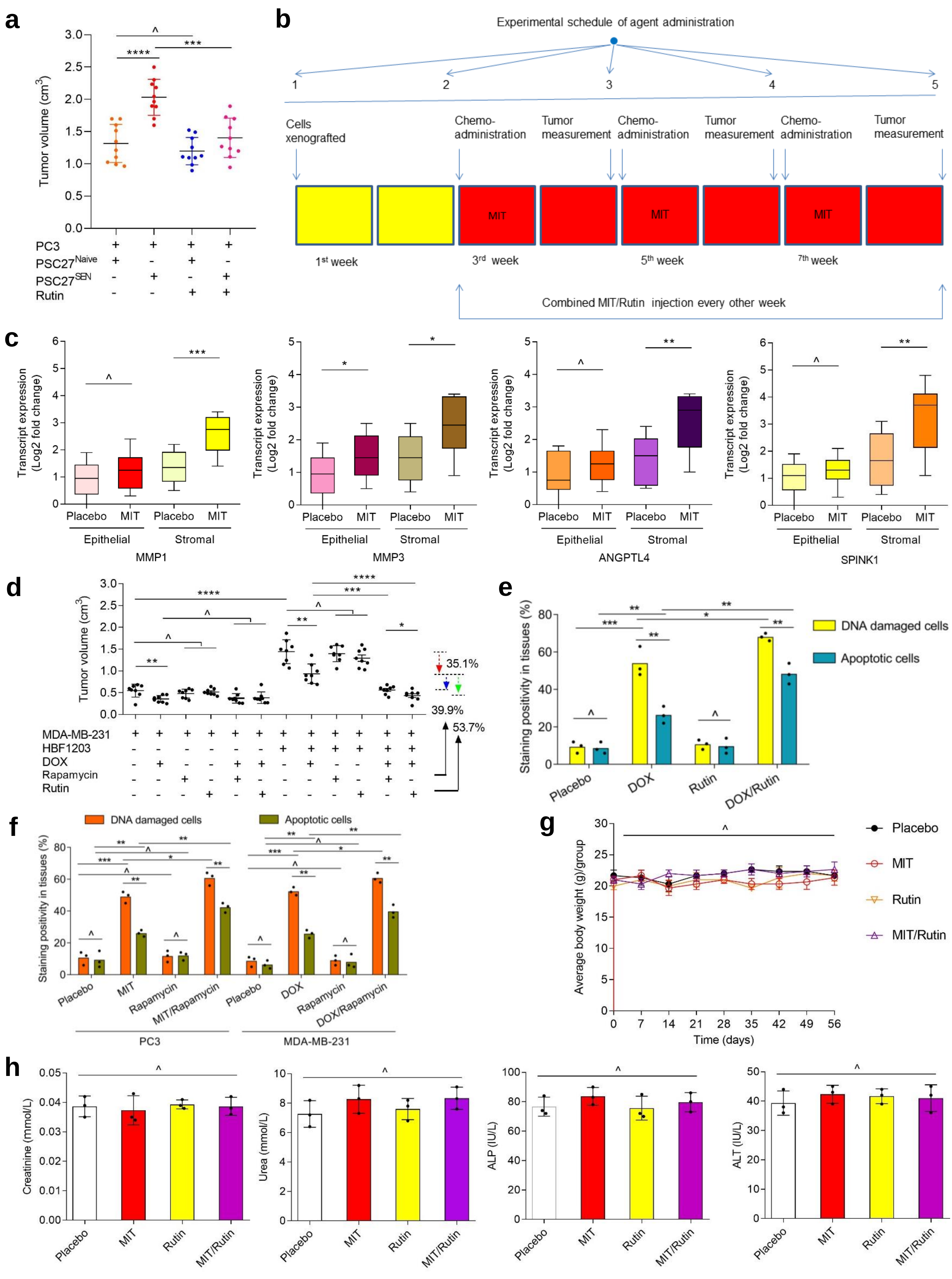

a
Tumor volume (cm3)
PC3
PSC27Naive
PSC27SEN
Rutin
b
Experimental schedule of agent administration
Cells xenografted
Chemo-administration
Tumor measurement
MIT
1st week
3rd week
5th week
7th week
Combined MIT/Rutin injection every other week
c
Transcript expression (Log2 fold change)
Placebo
MIT
Epithelial
Stromal
MMP1
MMP3
ANGPTL4
SPINK1
d
Tumor volume (cm3)
MDA-MB-231
HBF1203
DOX
Rapamycin
Rutin
35.1%
39.9%
53.7%
e
Staining positivity in tissues (%)
DNA damaged cells
Apoptotic cells
Placebo
DOX
Rutin
DOX/Rutin
f
DNA damaged cells
Apoptotic cells
Staining positivity in tissues (%)
Placebo
MIT
Rapamycin
MIT/Rapamycin
DOX
DOX/Rapamycin
PC3
MDA-MB-231
g
Average body weight (g)/group
Time (days)
Placebo
MIT
Rutin
MIT/Rutin
h
Creatinine (mmol/L)
Urea (mmol/L)
ALP (IU/L)
ALT (IU/L)
Placebo
MIT
Rutin
MIT/Rutin

Figure S7

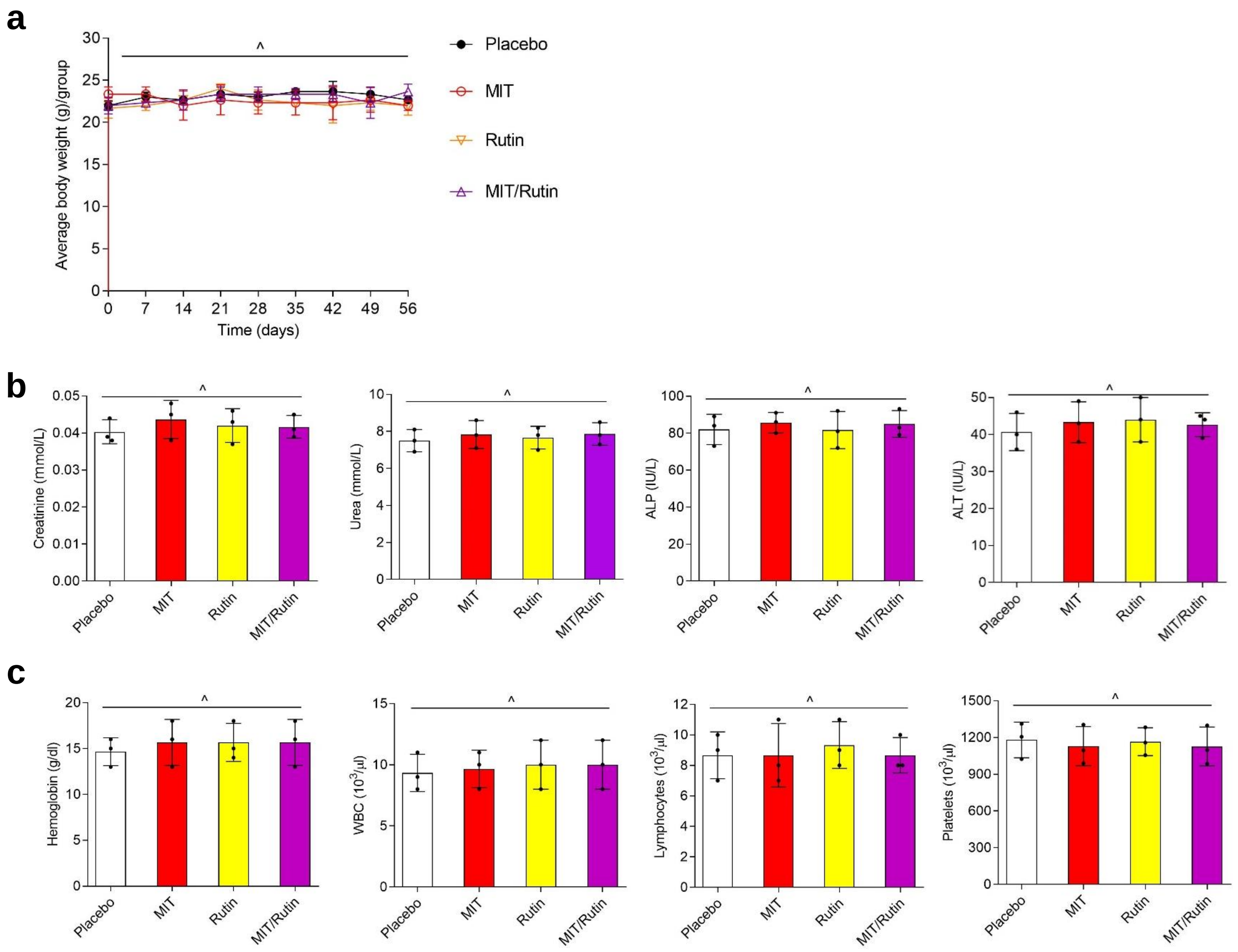

a
Average body weight (g)/group
Time (days)
Placebo
MIT
Rutin
MIT/Rutin
b
Creatinine (mmol/L)
Urea (mmol/L)
ALP (IU/L)
ALT (IU/L)
c
Hemoglobin (g/dl)
WBC (10³/µl)
Lymphocytes (10³/µl)
Platelets (10³/µl)

Supplementary Figure Legends

Figure S1. Characterization of therapy-induced senescence, senolyitcs screening and phytochemical profiling of the senomorphic candidate rutin.

(**a**) Representative images that show SA- β-Gal staining results after treatment of PSC27 cells with CTRL (DMSO) or BLEO in culture. Scale bar, 20 μm. (**b**) Representative images that display BrdU staining profiles of PSC27 cell populations treated with CTRL or BLEO. (**c**) Comparative statistics of immunofluorescence staining of γH2AX to probe DNA damage response (DDR) intensity. The DDR profile was classified into 4 sub-categories including 0 foci, 1~3 foci, 4~10 foci and > 10 foci *per* cell. (**d**) Evaluation of the effects of individual agents (herein group A) of the NMA library on the survival of CTRL and SEN cells in culture. (**e**) A chemical formula of the natural flavonoid rutin. (**f**) High resolution mass spectra exhibiting the total ion chromatogram (TIC, top) and base peak chromatogram (BPC, bottom) of rutin (chemical pure product) after performance of HPLC-ESI-QTOF-MS. CTRL, control. BLEO, bleomycin. Data in **a**-**d** are representative of 3 independent experiments. *P* values were calculated by a two-sided *t*-test. ***, $P < 0.001$. ****, $P < 0.0001$.

Figure S2. Overall profiling of transcriptome-wide expression of senescent stromal cells upon exposure to rutin in the culture.

(**a**) Heatmap depicting the expression landscape of PSC27 senescent cells and their counterparts exposed to rutin (100 μM). Note there were 3733 and 886 genes, expression of which was significantly downregulated and upregulated by rutin, respectively. (**b**-**c**) Pie charts depicting the cellular components (CC) (**b**) and biological processes (BP) (**c**) of top 100 genes most downregulated by rutin as revealed by GO analysis. (**d**) Quantitative analysis of representative SASP factor expression at transcription level in PSC27 upon replicative senescence (RS) in the absence or presence of rutin (at increasing concentrations) in culture. (**e**) Quantitative analysis of representative SASP factor expression at transcription level in PSC27 upon oncogene (*HRas*^{G12V})-induced senescence (OIS) in the absence or presence of rutin (at increasing concentrations) in culture. (**f**) Quantitative analysis of representative SASP factor expression at transcription level in WI38 upon BLEO-induced senescence in the absence or presence of rutin (at increasing concentrations) in culture. (**g**) Quantitative assessment of representative SASP factor expression at transcription level in IMR90 upon BLEO-induced senescence in the absence or presence of rutin (at increasing concentrations) in culture. IL2 and

IL12, non-SASP inflammatory factors used as experimental controls. CTRL, control. BLEO, bleomycin. Data in **d**-**g** are representative of 3 independent experiments. *P* values were calculated by a two-sided *t*-test. ^, $P > 0.05$. *, $P < 0.05$. **, $P < 0.01$. ***, $P < 0.001$. ****, $P < 0.0001$.

Figure S3. Proteome-wide mapping of potential interactions between target proteins and other molecules.

(**a**) A BioGRID-based profiling of ATM-interactive molecules, using a biomedical interaction repository with data compiled and applied as a public database archiving and disseminating protein interaction data from human. (**b**) A BioGRID-based profiling of TRAF6-interactive molecules, with a biomedical interaction repository as described in (**a**). (**c**) Quantitative analysis of representative SASP factor expression at transcription level in PSC27 upon BLEO-induced senescence in the absence or presence of rutin, VH298 and/or IL17A in culture. VH298 and IL17A, HIF1α and TRAF6 activators, respectively. CTRL, control. BLEO, bleomycin. Data in **c** are representative of 3 independent experiments. *P* values were calculated by a two-sided *t*-test. ^, $P > 0.05$. *$P < 0.05$. **$P < 0.01$. ***$P < 0.001$. ****$P < 0.0001$.

Figure S4. Characterization of prostate cancer cell malignancy upon short time treatment with conditioned media of stromal cells and experimental assays to profile the efficacy of rapamycin as a senomorphic agent.

(**a**) PCa cells were treated with the CM from PSC27 sublines as indicated for 24 h, and subject to cell proliferation assay. Native and senescent stromal cells generated by BLEO treatment were employed, with the CM collected 7 d after drug treatment and used for PCa cell culture (PC3, DU145, M12 and LNCaP). The CM were collected from equal number of cells *per* condition, with a starting DMEM that contains 0.5% FBS to make the CM. (**b**) PCa cells were treated with the CM from PSC27 sublines as indicated for 24 h, and subject to drug resistance assay. Stromal cells were processed, with their CM collected and applied for cancer cell-based assays as described in (**a**). (**c**) PCa cells were treated with the CM from PSC27 sublines as indicated for 3 d, and subject to cell proliferation assay. The senomorphic agent rapamycin was used to determine its effect on the proliferative capacity of cancer cells. (**d**) PCa cells were treated with the CM from PSC27 sublines as indicated for 3 d, and subject to cell migration assay. The senomorphic agent rapamycin was used to determine its effect on the migration activity of cancer cells. (**e**) PCa cells were treated with the CM from PSC27 sublines as indicated for

3 d, and subject to cell invasion assay. The senomorphic agent rapamycin was used to determine its effect on the invasiveness activity of cancer cells. (**f**) PCa cells were treated with the CM from PSC27 sublines as indicated for 3 d, and subject to drug resistance assay. The senomorphic agent rapamycin was used to determine its effect on the chemoresistance potential of cancer cells. PCa, prostate cancer. CM, conditioned media. CTRL, control. BLEO, bleomycin. Data in **a-f** are representative of 3 independent experiments. *P* values were calculated by a two-sided *t*-test. ^, $P > 0.05$; *$P < 0.05$; **$P < 0.01$; ***$P < 0.001$; ****$P < 0.0001$.

Figure S5. Characterization of breast cancer cell malignancy upon treatment with conditioned media of human stromal cells in the absence or presence of ruin.

(**a**) BCa cells were treated with the CM from HBF1203 sublines as indicated for 24 h, and subject to cell proliferation assay. Native and senescent stromal cells generated by DOX treatment were employed, with the CM collected 7 d after drug treatment and used for BCa cell culture (MDA-MB-231, SUM159, MCF7 and T47D). The CM were collected from an equal number of cells *per* condition, with a starting DMEM that contains 0.5% FBS to make the CM. (**b**) Migration assay of BCa cells described in (**a**) and seeded within trans-wells in 6-well plates, with cells cultured for 3 d in the CM from HBF1203 sublines depicted in (**a**). (**c**) Invasiveness measurement of BCa cells described in (**a**) across the trans-well membrane upon culture with the CM from HBF1203 sublines described in (**a**). (**d**) Chemoresistance assay of BCa cells described in (**a**) cultured with the CM from HBF1203 sublines described in (**a**). MIT (mitoxantrone) was applied at the concentration of IC50 value pre-determined *per* cell line. (**e**) Dose-response curves (non-linear regression/curve fit) plotted from drug-based survival assays of MDA-MB-231 cells cultured with the CM of HBF1203 native or induced senescent by DOX (HBF1203-DOX), and concurrently treated by a wide range of concentrations rutin. Data are representative of 3 independent experiments. BCa, breast cancer. CM, conditioned media. CTRL, control. DOX, doxorubicin. Data in **a-e** are representative of 3 independent experiments. *P* values were calculated by a two-sided *t*-test. ^, $P > 0.05$. *$P < 0.05$. **$P < 0.01$. ***$P < 0.001$. ****$P < 0.0001$.

Figure S6. Senescent stromal cells confer therapeutic resistance on solid tumors but subject to reversal by rutin or rapamycin in experimental animals.

(**a**) Statistics of tumor volume measured at the end of an 8-week growth period. PC3 cells were xenografted alone or together with PSC27 cells to the hind flank of SCID mice. Prior to implantation, PSC27 cells were naïve, or subject to senescence induction by BLEO treatment ($PSC27^{Naive}$ and $PSC27^{SEN}$, respectively). (**b**) A strategic workflow of drug administration and tumor monitoring in the preclinical trial. PC3 cells alone or combined with PSC27 cells were inoculated subcutaneously to SCID mice 2 weeks prior to the initiation of chemotherapy. The chemotherapeutic agent MIT was provided on the first day of each week starting from the 3rd week, then given every other week with a total number of 3 doses. The senomorphic agent rutin was given 12 h before each time of MIT delivery (totally 3 doses in the regimen). At the end of 8 weeks mice were sacrificed with tumor volume measured, and histologically analyzed. (**c**) Quantitative transcript analysis of a subset of canonical SASP factors expressed in stromal cells isolated from the tumors of SCID mice. Tissues from animals implanted with both stromal and cancer cells in tumor grafts were subject to laser capture microdissection (LCM) isolation, RNA preparation and qRT-PCR assays. (**d**) Statistical comparison of tumor growth in animals after treatment by different agents. Mice received breast cancer cells (MDA-MB-231) alone or combined with breast stromal cells (HBF1203). Mice were treated by the chemotherapeutic drug DOX only or together with rutin. Tumor volumes were measured at the end of an 8-week preclinical regimen. (**e**) Statistical assessment of DNA-damaged and apoptotic cells in the tumor specimens analyzed in (**d**). Values are presented as percentage of cells positively stained by IHC with antibodies against γH2AX or caspase 3 (cleaved). (**f**) Statistical evaluation of DNA-damaged and apoptotic cells in the tumor specimens collected from both PCa (PC3) and BCa (MDA-MB-231) preclinical trials. Values are presented as percentage of cells positively stained by IHC with antibodies against γH2AX or caspase 3 (cleaved). Note, rapamycin was used as a senomorphic agent in these *in vivo* assays. (**g**) Mouse body weights were determined once a week until the end of the therapeutic regimen. Chemotherapeutic agent MIT (0.2 mg/kg) was administered alone or with the senomorphic agent rutin (10.0 mg/kg) on 1st day of week 3, 5 and 7 after tumor implantation (PC3/PSC27) to SCID mice. (**h**) Terminal bleeds were taken *via* cardiac punctures on day 56. Serum levels of creatinine, urea, alkaline phosphatase (ALP) and alanine aminotransferase (ALT) were analyzed for toxicity appraisal with SCID mice developing prostate tumors. Data are shown as mean ± SD and representative of 3 independent experiments. N = 10 per treatment arm. MIT,

mitoxantrone. DOX, doxorubicin. *P* values were calculated by Student's *t*- test (**a**, **c-h**). ^, $P > 0.05$. *$P < 0.05$. **$P < 0.01$. ***$P < 0.001$. ****$P < 0.0001$.

Figure S7. Chemotherapeutic and/or senomorphic agents generate negligible effects on body weight, biochemistry and blood counts of wildtype experimental mice (immunocompetent).

(**a**) Mouse body weights were determined once a week until the end of a therapeutic regimen applied to immunocompetent animals. Chemotherapeutic agent MIT (0.2 mg/kg) was administered alone or with the senomorphic agent rutin (10.0 mg/kg) on 1st day of week 3, 5 and 7 to wild type C57BL/6 mice. (**b**) Terminal bleeds were taken *via* cardiac punctures on the last day of the regime. Serum levels of creatinine, urea, alkaline phosphatase (ALP) and alanine aminotransferase (ALT) were analyzed for potential toxicity to C57BL/6 mice. (**c**) Blood counts were measured to evaluate potential effect of therapeutic agents on the immune system and tissue homeostasis of C57BL/6. WBC, white blood count. Data are shown as mean ± SD and representative of 3 independent experiments. N = 3 per treatment arm. MIT, mitoxantrone. *P* values were calculated by Student's *t*-test (**a-c**) (^, $P > 0.05$).

Table S1. A list of natural products in the NMA library and subject to screening for candidates of senotherapeutics.

No.	***Agent name***	***Molecular format***	***CAS no.***	***Cat. No.***
1	Icariin	$C_{33}H_{40}O_{15}$	489-32-7	S27246
2	Berberine	$C_{20}H_{18}NO_4+$	2086-83-1	S27357
3	Dandelion extract	NA	NA	S27201
4	Hedyotis diffusa wild extract	NA	NA	S27202
5	Coix seed extract	NA	NA	S27205
6	Salidroside extract	NA	NA	B20504
7	Gynostemma plant extract	$C_{80}H_{126}O_{44}$	15588-68-8	S33058
8	Cytisus scoparius (Scotch broom) extract	NA	84696-48-0	Y43750
9	Secoisolariciresinol diglucoside	$C_{32}H_{46}O_{16}$	148244-82-0	B21419
10	Acerola cherry extract	NA	NA	S30954
11	Ox bile powder	NA	8008-63-7	S31352
12	Andrographis extract	NA	90244-84-1	S25350
13	Gymnema sylvestre extract	NA	90045-47-9	S27074
14	Malt extract	NA	8002-48-0	S50858
15	Valerian root extract	NA	NA	S68176
16	Glipizide	$C_{21}H_{27}N_5O_4S$	29094-61-9	S28259
17	Alkannin	$C_{16}H_{16}O_5$	517-88-4	B50783
18	Ginkgo biloba extract	NA	NA	S31354
19	Rutin	$C_{27}H_{30}O_{16}$	153-18-4	S13033
20	Aloe vera	$C_{16}H_{13}NO_3$	85507-69-3	S27799
21	Ginger extract	$C_{21}H_{34}O_4$	84696-15-1	B73079
22	Yeast extract	NA	NA	MLP0021B
23	L-Arginine 2-oxopentanedioate	$C_{17}H_{38}N_8O_{11}$	5256-76-8	Y75565

24	Blueberry extract	NA	84082-34-8	S27668
25	Cucumis melon extract	NA	90063-94-8	B73460
26	Eucalyptus globulus extract	NA	84625-32-1	T63141
27	Bergenia purpurascens extract	NA	NA	S27422
28	Cardamom extract	NA	NA	S27640
29	Fructus corni extract	NA	NA	S27765
30	Radix Puerariae extract	NA	NA	S27789
31	Tribulus terrestris extract	NA	NA	S27823
32	Fructus cnidii	NA	NA	S25344
33	Red tea extract	NA	NA	S25883
34	Astragalus mongholicus extract			S27211
35	4-Carboxyphenylboronic acid	$C_7H_7BO_4$	14047-29-1	S42511
36	Pigeon liver extract	NA	NA	S29761
37	Licorice powder	NA	NA	S69134

Table S2. A list of candidate molecules with putative potential to interact with human ATM and/or TRAF6

(1) Interactors of ATM		*(2) Interactors of TRAF6*		*(3) Co-interactors of ATM and TRAF6*		*Effective inhibitors*
ABL1	*H. sapiens*	ABCF1	*H. sapiens*	AKIRIN2	*H. sapiens*	NA
ACTL6B	*H. sapiens*	AIMP2	*H. sapiens*	ATM	*H. sapiens*	KU55933, ATM-IN-1, AZ32
ACVR1	*H. sapiens*	AJUBA	*H. sapiens*	BCL10	*H. sapiens*	Bcl10 peptide inhibitors (BPIs)
AKIRIN2	*H. sapiens*	AKIRIN2	*H. sapiens*	BSG	*H. sapiens*	MiR-146a-5p
ALB	*H. sapiens*	AKT1	*H. sapiens*	CBL	*H. sapiens*	NA
AMIGO1	*H. sapiens*	ALDH1B1	*H. sapiens*	CHUK/IKKα	*H. sapiens*	NA
AP1B1	*H. sapiens*	AMBRA1	*H. sapiens*	H2AFX	*H. sapiens*	NA
AP3B1	*H. sapiens*	APP	*H. sapiens*	HIF1A	*H. sapiens*	LW6, HIF-1α-IN-2, CDMP-TQZ, PX-478, Echinomycin
APEX1	*H. sapiens*	APPL1	*H. sapiens*	IKBKB/IKKβ	*H. sapiens*	IKK2-IN-3, PF-184, AZD3264, BOT-64
APLNR	*H. sapiens*	ARRB1	*H. sapiens*	IKBKG/IKKγ	*H. sapiens*	NA
ATF2	*H. sapiens*	ARRB2	*H. sapiens*	MAP3K7/TAK1	*H. sapiens*	Takinib, 5Z-7, TAK1-IN-2
ATG16L1	*H. sapiens*	ASB17	*H. sapiens*	NOD2	*H. sapiens*	NOD-IN-1, GSK717
ATM	*H. sapiens*	ASXL2	*H. sapiens*	NPM1	*H. sapiens*	NSC348884
ATMIN	*H. sapiens*	ATM	*H. sapiens*	PARK2	*H. sapiens*	NA
ATR	*H. sapiens*	ATP1B1	*H. sapiens*	PELI1	*H. sapiens*	NA
ATRIP	*H. sapiens*	ATXN3	*H. sapiens*	RANBP9	*H. sapiens*	NA
AVEN	*H. sapiens*	BANK1	*H. sapiens*	SOCS1	*H. sapiens*	Suppressors of cytokine signaling 1
AVPR2	*H. sapiens*	BCL10	*H. sapiens*	SPOP	*H. sapiens*	SPOP-IN-6b, NEO2734
BAG6	*H. sapiens*	BCL3	*H. sapiens*	TP53	*H. sapiens*	NA
BANP	*H. sapiens*	BECN1	*H. sapiens*	TRAF6	*H. sapiens*	NA
BAZ1A	*H. sapiens*	BIRC2	*H. sapiens*	TRIM29	*H. sapiens*	NA
BAZ1B	*H. sapiens*	BIRC3	*H. sapiens*	TRIM37	*H. sapiens*	NA
BCAS3	*H. sapiens*	BMPR1B	*H. sapiens*			
BCL10	*H. sapiens*	BRSK2	*H. sapiens*			
BHLHE41	*H. sapiens*	BSG	*H. sapiens*			
BLM	*H. sapiens*	CALCOCO2	*H. sapiens*			
BMI1	*H. sapiens*	CANX	*H. sapiens*			
BRAT1	*H. sapiens*	CARD11	*H. sapiens*			
BRCA1	*H. sapiens*	CARS	*H. sapiens*			
BRCA2	*H. sapiens*	CASP4	*H. sapiens*			
BRD4	*H. sapiens*	CASP8	*H. sapiens*			
BSG	*H. sapiens*	CBL	*H. sapiens*			
C1ORF27	*H. sapiens*	CBLB	*H. sapiens*			
C5AR1	*H. sapiens*	CCDC53	*H. sapiens*			
C5AR2	*H. sapiens*	CCDC67	*H. sapiens*			
CBL	*H. sapiens*	CD40	*H. sapiens*			
CD274	*H. sapiens*	CHUK	*H. sapiens*			
CD70	*H. sapiens*	COG6	*H. sapiens*			
CD80	*H. sapiens*	CPN2	*H. sapiens*			
CD99L2	*H. sapiens*	CRBN	*H. sapiens*			
CDC6	*H. sapiens*	CRTC2	*H. sapiens*			
CDK9	*H. sapiens*	CSNK1G1	*H. sapiens*			
CDKN2C	*H. sapiens*	CTNNB1	*H. sapiens*			
CHD4	*H. sapiens*	CTPS1	*H. sapiens*			
CHEK1	*H. sapiens*	CUL5	*H. sapiens*			
CHEK2	*H. sapiens*	CYCS	*H. sapiens*			
CHUK	*H. sapiens*	CYLD	*H. sapiens*			
COPS5	*H. sapiens*	DCBLD2	*H. sapiens*			
CREB1	*H. sapiens*	DCP1A	*H. sapiens*			
CRX	*H. sapiens*	DDB1	*H. sapiens*			
CSNK1D	*H. sapiens*	DDX17	*H. sapiens*			
CTDP1	*H. sapiens*	DDX39A	*H. sapiens*			
CXCR3	*H. sapiens*	DDX3X	*H. sapiens*			
CXCR4	*H. sapiens*	DHX58	*H. sapiens*			

CXXC5	*H. sapiens*
CYB5B	*H. sapiens*
DAXX	*H. sapiens*
DCAF13	*H. sapiens*
DCLRE1A	*H. sapiens*
DCLRE1C	*H. sapiens*
DDX1	*H. sapiens*
DDX58	*H. sapiens*
DNAJC7	*H. sapiens*
DRD4	*H. sapiens*
DYRK2	*H. sapiens*
E2F1	*H. sapiens*
E4F1	*H. sapiens*
EDEM1	*H. sapiens*
EDNRB	*H. sapiens*
EFNA4	*H. sapiens*
EIF3E	*H. sapiens*
EIF4EBP1	*H. sapiens*
ELAVL1	*H. sapiens*
EP400	*H. sapiens*
ERRFI1	*H. sapiens*
ESR2	*H. sapiens*
ESRRG	*H. sapiens*
EXO1	*H. sapiens*
FANCD2	*H. sapiens*
FANCI	*H. sapiens*
FECH	*H. sapiens*
FEN1	*H. sapiens*
FOXO1	*H. sapiens*
FOXO3	*H. sapiens*
FPR1	*H. sapiens*
GAPDHS	*H. sapiens*
GCGR	*H. sapiens*
GLTSCR2	*H. sapiens*
GMNN	*H. sapiens*
GNA11	*H. sapiens*
GPR17	*H. sapiens*
GPR182	*H. sapiens*
GPR45	*H. sapiens*
GPR64	*H. sapiens*
GTR6	*H. sapiens*
H2AFX	*H. sapiens*
HCST	*H. sapiens*
HDAC1	*H. sapiens*
HDAC6	*H. sapiens*
HIF1A	*H. sapiens*
HIST1H3A	*H. sapiens*
HLA-A	*H. sapiens*
HNRNPUL1	*H. sapiens*
HSPA8	*H. sapiens*
HUWE1	*H. sapiens*
HYPM	*H. sapiens*
IGHM	*H. sapiens*
IK	*H. sapiens*
IKBKB	*H. sapiens*
IKBKG	*H. sapiens*
IL24	*H. sapiens*
DLG4	*H. sapiens*
DNA2	*H. sapiens*
DNAJC5	*H. sapiens*
ECSIT	*H. sapiens*
EDA2R	*H. sapiens*
EDARADD	*H. sapiens*
EEF1A1	*H. sapiens*
EIF2AK2	*H. sapiens*
EIF4E2	*H. sapiens*
EMILIN1	*H. sapiens*
EPPK1	*H. sapiens*
ERN1	*H. sapiens*
ESR1	*H. sapiens*
EZH2	*H. sapiens*
FAM177A1	*H. sapiens*
FANCA	*H. sapiens*
FGG	*H. sapiens*
FHL2	*H. sapiens*
FOXG1	*H. sapiens*
FUS	*H. sapiens*
FYN	*H. sapiens*
GANAB	*H. sapiens*
GART	*H. sapiens*
GNB2L1	*H. sapiens*
GSK3B	*H. sapiens*
GTF2I	*H. sapiens*
H2AFX	*H. sapiens*
HDAC3	*H. sapiens*
HIF1A	*H. sapiens*
HIST2H2BE	*H. sapiens*
HNRNPA1	*H. sapiens*
HSD17B10	*H. sapiens*
HSPA4	*H. sapiens*
HSPB2	*H. sapiens*
HTT	*H. sapiens*
IARS	*H. sapiens*
ID1	*H. sapiens*
IFI16	*H. sapiens*
IFIT3	*H. sapiens*
IFNLR1	*H. sapiens*
IKBKB	*H. sapiens*
IKBKE	*H. sapiens*
IKBKG	*H. sapiens*
IL17A	*H. sapiens*
IL17RA	*H. sapiens*
IL1R1	*H. sapiens*
IL1RL1	*H. sapiens*
IPMK	*H. sapiens*
IQUB	*H. sapiens*
IRAK1	*H. sapiens*
IRAK2	*H. sapiens*
IRAK3	*H. sapiens*
IRAK4	*H. sapiens*
IRF1	*H. sapiens*
IRF3	*H. sapiens*
IRF4	*H. sapiens*
IRF5	*H. sapiens*

IL6R	*H. sapiens*	IRF7	*H. sapiens*
ITFG3	*H. sapiens*	IRF8	*H. sapiens*
KAT5	*H. sapiens*	ITCH	*H. sapiens*
KAT8	*H. sapiens*	JAK2	*H. sapiens*
KDM2A	*H. sapiens*	KCNQ1	*H. sapiens*
KDM6A	*H. sapiens*	KDM4B	*H. sapiens*
KIAA1429	*H. sapiens*	KIR2DL4	*H. sapiens*
KPNA1	*H. sapiens*	KLF4	*H. sapiens*
KPNA3	*H. sapiens*	KMT2E	*H. sapiens*
KPNA5	*H. sapiens*	KRAS	*H. sapiens*
KPNB1	*H. sapiens*	KRT1	*H. sapiens*
LIG4	*H. sapiens*	KRT10	*H. sapiens*
LMNA	*H. sapiens*	KRT12	*H. sapiens*
LPAR4	*H. sapiens*	KRT2	*H. sapiens*
LPAR6	*H. sapiens*	KRT3	*H. sapiens*
LRRC25	*H. sapiens*	KRT5	*H. sapiens*
MAGEC2	*H. sapiens*	KRT8	*H. sapiens*
MAP1S	*H. sapiens*	KRT9	*H. sapiens*
MAP3K4	*H. sapiens*	KXD1	*H. sapiens*
MAP3K7	*H. sapiens*	LAT	*H. sapiens*
MAPK1	*H. sapiens*	LGALS3BP	*H. sapiens*
MAPK14	*H. sapiens*	LGMN	*H. sapiens*
MAPK8	*H. sapiens*	LIMD1	*H. sapiens*
MAS1	*H. sapiens*	LNX1	*H. sapiens*
MCM2	*H. sapiens*	LRP5	*H. sapiens*
MCPH1	*H. sapiens*	LRP6	*H. sapiens*
MDC1	*H. sapiens*	MAGEE2	*H. sapiens*
MDM2	*H. sapiens*	MALT1	*H. sapiens*
MED1	*H. sapiens*	MAP1LC3A	*H. sapiens*
MINK1	*H. sapiens*	MAP1LC3B	*H. sapiens*
MLH1	*H. sapiens*	MAP2K6	*H. sapiens*
MLNR	*H. sapiens*	MAP3K11	*H. sapiens*
MRE11A	*H. sapiens*	MAP3K14	*H. sapiens*
MSH2	*H. sapiens*	MAP3K3	*H. sapiens*
MSH6	*H. sapiens*	MAP3K5	*H. sapiens*
MTA3	*H. sapiens*	MAP3K7	*H. sapiens*
MTMR3	*H. sapiens*	MAP3K8	*H. sapiens*
MTRR	*H. sapiens*	MAPT	*H. sapiens*
NAT2	*H. sapiens*	MARCH5	*H. sapiens*
NBN	*H. sapiens*	MAST2	*H. sapiens*
ND4	*H. sapiens*	MATR3	*H. sapiens*
NDUFA8	*H. sapiens*	MAVS	*H. sapiens*
NFE2L2	*H. sapiens*	MCCC1	*H. sapiens*
NHEJ1	*H. sapiens*	MCL1	*H. sapiens*
NKX3-1	*H. sapiens*	MCM7	*H. sapiens*
NMUR1	*H. sapiens*	MEOX2	*H. sapiens*
NOD2	*H. sapiens*	MSR1	*H. sapiens*
NOLC1	*H. sapiens*	MTHFD1	*H. sapiens*
NPM1	*H. sapiens*	MTOR	*H. sapiens*
NPTN	*H. sapiens*	MTURN	*H. sapiens*
NPY2R	*H. sapiens*	MUS81	*H. sapiens*
NR4A1	*H. sapiens*	MVP	*H. sapiens*
NRD1	*H. sapiens*	MYB	*H. sapiens*
NREP	*H. sapiens*	MYD88	*H. sapiens*
OCIAD1	*H. sapiens*	NASP	*H. sapiens*
OPALIN	*H. sapiens*	NEAT1	*H. sapiens*
OPALIN	*H. sapiens*	NF1	*H. sapiens*

OPRM1	*H. sapiens*	NFATC2IP	*H. sapiens*
ORF7A	*H. sapiens*	NGFR	*H. sapiens*
OSGIN1	*H. sapiens*	NLRC3	*H. sapiens*
P2RY10	*H. sapiens*	NLRX1	*H. sapiens*
P3H2	*H. sapiens*	NOD2	*H. sapiens*
PAN2	*H. sapiens*	NOL3	*H. sapiens*
PARK2	*H. sapiens*	NPM1	*H. sapiens*
PARP1	*H. sapiens*	NR2C2	*H. sapiens*
PCNA	*H. sapiens*	NROB2	*H. sapiens*
PELI1	*H. sapiens*	NTRK1	*H. sapiens*
PER3	*H. sapiens*	NTRK2	*H. sapiens*
PEX5	*H. sapiens*	NUMBL	*H. sapiens*
PIM1	*H. sapiens*	OTUB1	*H. sapiens*
POLR2A	*H. sapiens*	OTUB2	*H. sapiens*
PPP1CA	*H. sapiens*	OTUD7A	*H. sapiens*
PPP1CC	*H. sapiens*	OTUD7B	*H. sapiens*
PPP2CA	*H. sapiens*	PAICS	*H. sapiens*
PPP2R4	*H. sapiens*	PARK2	*H. sapiens*
PPP2R5C	*H. sapiens*	PARK7	*H. sapiens*
PRKCA	*H. sapiens*	PDLIM7	*H. sapiens*
PRKDC	*H. sapiens*	PEBP1	*H. sapiens*
PRMT8	*H. sapiens*	PELI1	*H. sapiens*
PTCH1	*H. sapiens*	PELI2	*H. sapiens*
PTH1R	*H. sapiens*	PELI3	*H. sapiens*
PTH2R	*H. sapiens*	PFAS	*H. sapiens*
RAD17	*H. sapiens*	PFN1	*H. sapiens*
RAD50	*H. sapiens*	PHLDA1	*H. sapiens*
RAD51	*H. sapiens*	PIK3CA	*H. sapiens*
RANBP9	*H. sapiens*	PINK1	*H. sapiens*
RAP2A	*H. sapiens*	PLEKHF2	*H. sapiens*
RASSF1	*H. sapiens*	PLEKHO1	*H. sapiens*
RBBP8	*H. sapiens*	POLI	*H. sapiens*
RBM47	*H. sapiens*	PPP1CC	*H. sapiens*
RELA	*H. sapiens*	PPP4C	*H. sapiens*
RFC1	*H. sapiens*	PPP4R1	*H. sapiens*
RHEB	*H. sapiens*	PRDX1	*H. sapiens*
RNF123	*H. sapiens*	PRKAA1	*H. sapiens*
RNF20	*H. sapiens*	PRKACB	*H. sapiens*
RNF40	*H. sapiens*	PRKCD	*H. sapiens*
RPA1	*H. sapiens*	PRKCZ	*H. sapiens*
RPA2	*H. sapiens*	PRNP	*H. sapiens*
RPL11	*H. sapiens*	PSEN1	*H. sapiens*
RPL5	*H. sapiens*	PSEN2	*H. sapiens*
RPS6KA1	*H. sapiens*	PSMB5	*H. sapiens*
RPS6KA3	*H. sapiens*	PSMC1	*H. sapiens*
RRM2B	*H. sapiens*	PSMC2	*H. sapiens*
RSF1	*H. sapiens*	PSMC3	*H. sapiens*
SCN2B	*H. sapiens*	PSMC5	*H. sapiens*
SETD2	*H. sapiens*	PSMD1	*H. sapiens*
SIM2	*H. sapiens*	PSMD12	*H. sapiens*
SIRT7	*H. sapiens*	PSMD13	*H. sapiens*
SKP2	*H. sapiens*	PSMD3	*H. sapiens*
SLC22A4	*H. sapiens*	PSMD7	*H. sapiens*
SMAD7	*H. sapiens*	PTPN11	*H. sapiens*
SMARCA4	*H. sapiens*	PTPN6	*H. sapiens*
SMARCA5	*H. sapiens*	PYCARD	*H. sapiens*
SMARCB1	*H. sapiens*	RAD23A	*H. sapiens*

SMC1A	*H. sapiens*
SMC3	*H. sapiens*
SMO	*H. sapiens*
SMURF2	*H. sapiens*
SNAI1	*H. sapiens*
SNW1	*H. sapiens*
SNX6	*H. sapiens*
SOCS1	*H. sapiens*
SPOP	*H. sapiens*
SPSB1	*H. sapiens*
SREBF1	*H. sapiens*
SRSF8	*H. sapiens*
STAU1	*H. sapiens*
STK11	*H. sapiens*
TAF1	*H. sapiens*
TAOK3	*H. sapiens*
TCL1A	*H. sapiens*
TDP1	*H. sapiens*
TELO2	*H. sapiens*
TERF1	*H. sapiens*
TERF2	*H. sapiens*
TFF1	*H. sapiens*
TOP1	*H. sapiens*
TOP2B	*H. sapiens*
TOPBP1	*H. sapiens*
TP53	*H. sapiens*
TP53BP1	*H. sapiens*
TPSAB1	*H. sapiens*
TPT1	*H. sapiens*
TRAF6	*H. sapiens*
TRIM28	*H. sapiens*
TRIM29	*H. sapiens*
TRIM37	*H. sapiens*
TTI1	*H. sapiens*
UCHL3	*H. sapiens*
UCK1	*H. sapiens*
UNC93B1	*H. sapiens*
USP28	*H. sapiens*
USP34	*H. sapiens*
USP37	*M. musculus*
UTP15	*H. sapiens*
VHL	*H. sapiens*
VPRBP	*H. sapiens*
WHSC1L1	*H. sapiens*
WRN	*H. sapiens*
WWOX	*H. sapiens*
XPA	*H. sapiens*
XPC	*H. sapiens*
XRCC5	*H. sapiens*
YWHAZ	*H. sapiens*
ZBTB48	*H. sapiens*
ZEB1	*H. sapiens*
ZER1	*H. sapiens*
ZNF821	*H. sapiens*

RANBP9	*H. sapiens*
RBCK1	*H. sapiens*
RCAN1	*H. sapiens*
RIPK2	*H. sapiens*
RNF114	*H. sapiens*
RNF152	*H. sapiens*
RNF166	*H. sapiens*
RNF31	*H. sapiens*
RPL3	*H. sapiens*
RPS2	*H. sapiens*
RPS20	*H. sapiens*
RPS27A	*H. sapiens*
RPTOR	*H. sapiens*
RRAGC	*H. sapiens*
RSAD2	*H. sapiens*
RUNX1	*H. sapiens*
RUSC1	*H. sapiens*
RXRA	*H. sapiens*
S100P	*H. sapiens*
SARM1	*H. sapiens*
SDCBP	*H. sapiens*
SERINC5	*H. sapiens*
SH3PXD2A	*H. sapiens*
SIGIRR	*H. sapiens*
SMAD4	*H. sapiens*
SMN1	*H. sapiens*
SMURF1	*H. sapiens*
SNAP29	*H. sapiens*
SNCA	*H. sapiens*
SOCS1	*H. sapiens*
SOCS3	*H. sapiens*
SPHK1	*H. sapiens*
SPOP	*H. sapiens*
SQSTM1	*H. sapiens*
SRC	*H. sapiens*
ST3GAL1	*H. sapiens*
STAMBP	*H. sapiens*
STAT3	*H. sapiens*
STAT6	*H. sapiens*
STK17A	*H. sapiens*
STK26	*H. sapiens*
STK4	*H. sapiens*
STRADB	*H. sapiens*
STUB1	*H. sapiens*
SYK	*H. sapiens*
TAB1	*H. sapiens*
TAB2	*H. sapiens*
TAB3	*H. sapiens*
TANK	*H. sapiens*
TARBP2	*H. sapiens*
TARS	*H. sapiens*
TAX1BP1	*H. sapiens*
TBK1	*H. sapiens*
TDP2	*H. sapiens*
TGFBR1	*H. sapiens*
TGFBR2	*H. sapiens*
TICAM1	*H. sapiens*

TICAM2	*H. sapiens*
TIFA	*H. sapiens*
TIRAP	*H. sapiens*
TJP2	*H. sapiens*
TLR3	*H. sapiens*
TLR4	*H. sapiens*
TLR9	*H. sapiens*
TMEM173	*H. sapiens*
TMEM189	*H. sapiens*
TMUB1	*H. sapiens*
TNF	*H. sapiens*
TNFAIP3	*H. sapiens*
TNFRSF11A	*H. sapiens*
TNFRSF13B	*H. sapiens*
TNFRSF17	*H. sapiens*
TNFRSF1A	*H. sapiens*
TNFRSF1B	*H. sapiens*
TNIK	*H. sapiens*
TOLLIP	*H. sapiens*
TOMM20	*H. sapiens*
TOMM70A	*H. sapiens*
TP53	*H. sapiens*
TRADD	*H. sapiens*
TRAF1	*H. sapiens*
TRAF2	*H. sapiens*
TRAF3IP1	*H. sapiens*
TRAF3IP2	*H. sapiens*
TRAF4	*H. sapiens*
TRAF5	*H. sapiens*
TRAF6	*H. sapiens*
TRAFD1	*H. sapiens*
TRAIP	*H. sapiens*
TRIM13	*H. sapiens*
TRIM17	*H. sapiens*
TRIM25	*H. sapiens*
TRIM29	*H. sapiens*
TRIM31	*H. sapiens*
TRIM37	*H. sapiens*
TRIM38	*H. sapiens*
TXNIP	*H. sapiens*
UBC	*H. sapiens*
UBE20	*H. sapiens*
UBE2D1	*H. sapiens*
UBE2D2	*H. sapiens*
UBE2D3	*H. sapiens*
UBE2D4	*H. sapiens*
UBE2E1	*H. sapiens*
UBE2I	*H. sapiens*
UBE2L3	*H. sapiens*
UBE2N	*H. sapiens*
UBE2V1	*H. sapiens*
UBL4A	*H. sapiens*
UBOX5	*H. sapiens*
UBTD1	*H. sapiens*
UBXN7	*H. sapiens*
UCHL1	*H. sapiens*
UEVLD	*H. sapiens*

ULK1	*H. sapiens*
USP1	*H. sapiens*
USP10	*H. sapiens*
USP14	*H. sapiens*
USP15	*H. sapiens*
USP17L9P	*H. sapiens*
USP19	*H. sapiens*
USP2	*H. sapiens*
USP20	*H. sapiens*
USP21	*H. sapiens*
USP3	*H. sapiens*
USP33	*H. sapiens*
USP39	*H. sapiens*
USP4	*H. sapiens*
USP5	*H. sapiens*
USP7	*H. sapiens*
VARS	*H. sapiens*
VAV3	*H. sapiens*
VCP	*H. sapiens*
VDAC1	*H. sapiens*
VPS52	*H. sapiens*
WDFY3	*H. sapiens*
WDR34	*H. sapiens*
WDR5	*H. sapiens*
WWP1	*H. sapiens*
XIAP	*H. sapiens*
YAP1	*H. sapiens*
YBX1	*H. sapiens*
YES1	*H. sapiens*
YOD1	*H. sapiens*
YWHAQ	*H. sapiens*
ZBTB25	*H. sapiens*
ZC3H12A	*H. sapiens*
ZDHHC11	*H. sapiens*
ZFAND5	*H. sapiens*
ZMYND11	*H. sapiens*
ZNF326	*H. sapiens*
ZNF675	*H. sapiens*
ZRANB1	*H. sapiens*
ZW10	*H. sapiens*

Note: molecules labelled in red are candidates that can likely interact with both ATM and TRAF6.
